# STAG2 promotes the myelination transcriptional program in oligodendrocytes

**DOI:** 10.7554/eLife.77848

**Published:** 2022-08-12

**Authors:** Ningyan Cheng, Guanchen Li, Mohammed Kanchwala, Bret M Evers, Chao Xing, Hongtao Yu

**Affiliations:** 1 Department of Pharmacology, University of Texas Southwestern Medical Center Dallas United States; 2 Westlake Laboratory of Life Sciences and Biomedicine Hangzhou China; 3 School of Life Sciences, Westlake University Hangzhou China; 4 Institute of Biology, Westlake Institute for Advanced Study Hangzhou China; 5 Eugene McDermott Center for Human Growth and Development, University of Texas Southwestern Medical Center Dallas United States; 6 Division of Neuropathology, University of Texas Southwestern Medical Center Dallas United States; 7 Department of Bioinformatics, Department of Population and Data Sciences, University of Texas Southwestern Medical Center Dallas United States; University of Edinburgh United Kingdom; Weill Cornell Medicine United States

**Keywords:** cohesin, loop extrusion, transcription, oligodendrocytes, Mouse

## Abstract

Cohesin folds chromosomes via DNA loop extrusion. Cohesin-mediated chromosome loops regulate transcription by shaping long-range enhancer–promoter interactions, among other mechanisms. Mutations of cohesin subunits and regulators cause human developmental diseases termed cohesinopathy. Vertebrate cohesin consists of SMC1, SMC3, RAD21, and either STAG1 or STAG2. To probe the physiological functions of cohesin, we created conditional knockout (cKO) mice with *Stag2* deleted in the nervous system. *Stag2* cKO mice exhibit growth retardation, neurological defects, and premature death, in part due to insufficient myelination of nerve fibers. *Stag2* cKO oligodendrocytes exhibit delayed maturation and downregulation of myelination-related genes. *Stag2* loss reduces promoter-anchored loops at downregulated genes in oligodendrocytes. Thus, STAG2-cohesin generates promoter-anchored loops at myelination-promoting genes to facilitate their transcription. Our study implicates defective myelination as a contributing factor to cohesinopathy and establishes oligodendrocytes as a relevant cell type to explore the mechanisms by which cohesin regulates transcription.

## Introduction

Chromosomes in a single human diploid cell, if linearly stitched together, span a length of more than 2 m. They need to be properly folded to be housed in the cell nucleus with a diameter of 10 µm. Chromosome folding occurs in a dynamic, structured way that regulates gene expression, and DNA replication and repair. Initially discovered as the molecular glue that tethers sister chromatids for segregation during mitosis (Haarhuis et al., 2014; Uhlmann, 2016; Yatskevich et al., 2019; Zheng and Yu, 2015), the cohesin complex has later been shown to be critical for structured chromosome folding and gene expression (Haarhuis et al., 2017; Rao et al., 2017; Schwarzer et al., 2017; Wutz et al., 2017).

Cohesin is loaded on chromosomes by the cohesin loader NIPBL. The cohesin–NIPBL complex can extrude DNA loops bidirectionally in an ATP-dependent manner (Davidson et al., 2019; Kim et al., 2019; Vian et al., 2018). The chromatin insulator CTCF has been proposed to block loop extrusion by cohesin, establishing topologically associated domains (TADs) and marking TAD boundaries. Chromatin interactions within each TAD are favored whereas inter-TAD interactions are disfavored. Thus, chromosome loops and TADs shape long-range cis–element interactions, such as promoter–enhancer interactions, thereby regulating transcription.

The vertebrate cohesin complex contains four core subunits: the SMC1–SMC3 heterodimeric ATPase, the kleisin subunit RAD21 that links the ATPase heads, and the HEAT-repeat protein STAG1 or STAG2. STAG1 and STAG2 bind to RAD21 in a mutually exclusive manner and create docking sites for several regulatory proteins, including CTCF (Hara et al., 2014; Li et al., 2020). STAG1 and STAG2 also interact with DNA and the SMC1–SMC3 hinge domains (Shi et al., 2020). STAG1 and STAG2 play redundant roles in sister-chromatid cohesion in cultured human cells, as both need to be simultaneously depleted to produce overt cohesion defects (Hara et al., 2014).

Mutations of NIPBL and cohesin subunits, including STAG2, result in human developmental diseases termed cohesinopathies, which affect multiple organs and systems (Remeseiro et al., 2013b; Soardi et al., 2017). In patients with cohesinopathies, mental retardation and neurological abnormalities caused by brain development defects are common (Piché et al., 2019). Dysregulation of gene transcription as a result of reduced cohesin functions has been suggested to underlie these developmental defects (De Koninck and Losada, 2016; Remeseiro et al., 2013a). In addition, several cohesin genes, including *STAG2*, are frequently mutated in a variety of human cancers (Martincorena and Campbell, 2015).

In this study, we deleted *Stag2* specifically in the nervous system in the mouse. The *Stag2* cKO mice exhibited deficient myelination. Loss of STAG2 delayed the maturation of oligodendrocytes and reduced chromosome loops in oligodendrocytes and impaired the transcription of myelination-related genes. Our findings establish the requirement for cohesin in proper gene expression in specific cell types and implicate defective myelination as a potential contributing factor to cohesinopathy.

## Results

### *Stag2* ablation in the nervous system causes growth retardation and neurological defects

**Figure 1.**
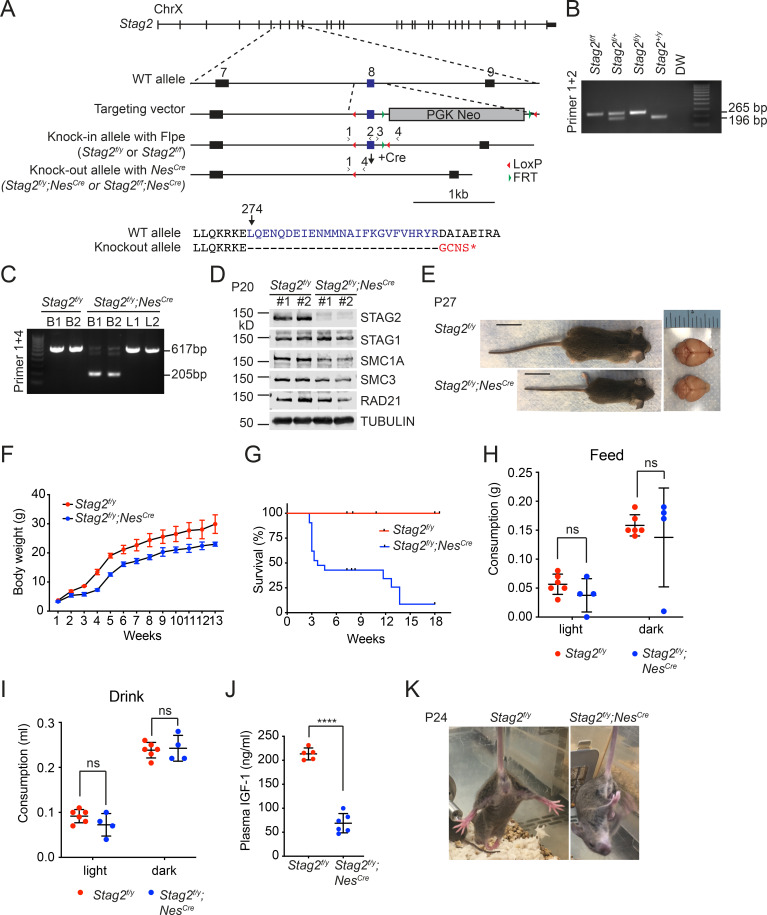
*Stag2* ablation in the mouse nervous system causes growth retardation and neurological defects. (**A**) Scheme for creating the ‘floxed’ *Stag2* allele by gene targeting. The genomic structure of the wild-type (WT) *Stag2* locus, the targeting vector, the knockin allele, the disrupted allele after Cre-mediated recombination, and the positions of the genotyping primers are shown. The amino acid sequence of the knockout allele in the targeted region is shown and aligned with that of the WT allele. (**B**) PCR analysis of the genomic DNA extracted from the tails of indicated mice with the primers in (**A**). (**C**) PCR analysis of genomic DNA extracted from brains (B) or livers (L) of indicated mice. (**D**) Immunoblots of brain lysates of *Stag2^f/y^* and *Stag2^f/y^;Nes^Cre^* mice with antibodies recognizing cohesin subunits and TUBULIN (as the loading control). (**E**) Representative images of *Stag2^f/y^* and *Stag2^f/y^;Nes^Cre^* mice. Scale bar = 2 cm. (**F**) Body weight of *Stag2^f/y^* and *Stag2^f/y^;Nes^Cre^* mice at different age. Mean ± standard deviation (SD) of at least three mice of the same age. (**G**) Survival curves of *Stag2^f/y^* (*n* = 12) and *Stag2^f/y^;Nes^Cre^* (*n* = 21) mice. Food (**H**) and water (**I**) consumption of 7- to 8-week-old *Stag2^f/y^* (*n* = 6) and *Stag2^f/y^;Nes^Cre^* (*n* = 4) mice. Mean ± SD; ns, not significant. (**J**) Plasma IGF-1 levels of 2-month-old *Stag2^f/y^* (*n* = 5) and *Stag2^f/y^;Nes^Cre^* (*n* = 6) mice. Mean ± SD; ****p < 0.0001. (**K**) Representative images of limb-clasping responses of *Stag2^f/y^* and *Stag2^f/y^;Nes^Cre^* mice. The uncropped images of blots in (**B–D**) are included in Figure 1—source data 1. Figure 1—source data 1.Uncropped images of gels and blots in Figure 1.

*Stag1* is required for mammalian embryonic development (Remeseiro et al., 2012), indicating that *Stag2* cannot compensate for the loss of *Stag1*. To examine the physiological functions of *Stag2* in the mouse, we created a *Stag2* ‘floxed’ mouse line (*Stag2^f/f^*) by homologous recombination with a template that contained two LoxP sites flanking exon 8 (Figure 1A, B) and targeted a critical exon (exon 8) of *Stag2*, which is located on the X chromosome, using CRISPR–Cas9 (Figure 1—figure supplement 1A). The *Stag2^null^* embryos showed severe developmental defects and underwent necrosis by E11.5 days (Figure 1—figure supplement 1B). Thus, *Stag2* is required for mouse embryonic development, consistent with a previous report (De Koninck et al., 2020). *Stag1* and *Stag2* have nonredundant developmental functions.

**Figure 1—figure supplement 1.**
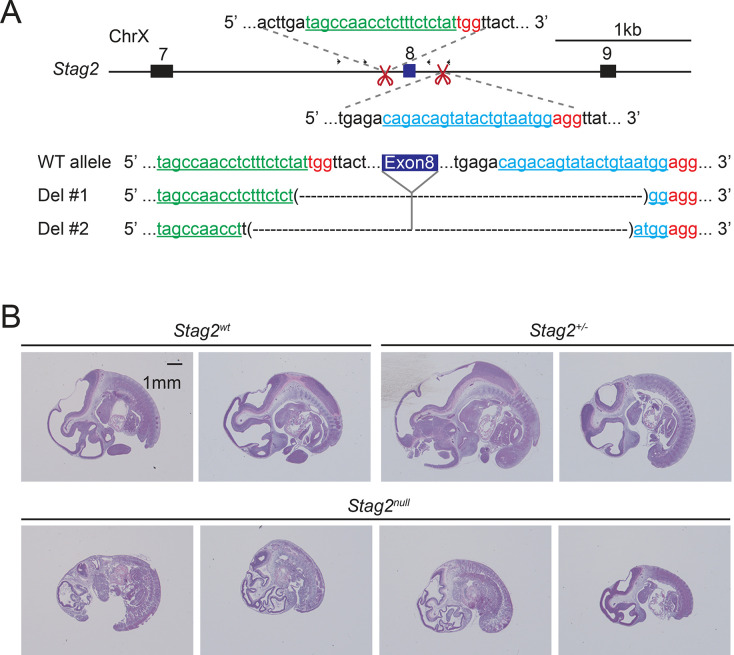
Generation of *Stag2* knockout mice using the CRISPR/Cas9 method. (**A**) Scheme for disrupting *Stag2* in the mouse genome using CRISPR/Cas9 with guide RNAs flanking exon 8. Sequencing analysis of the genomic DNA extracted from two *Stag2*-disrupted founder mice is shown below. (**B**) Hematoxylin and eosin (H&E) staining of sagittal sections of F2 embryos derived from F1 in (**A**) at E11.5.

**Figure 1—figure supplement 2.**
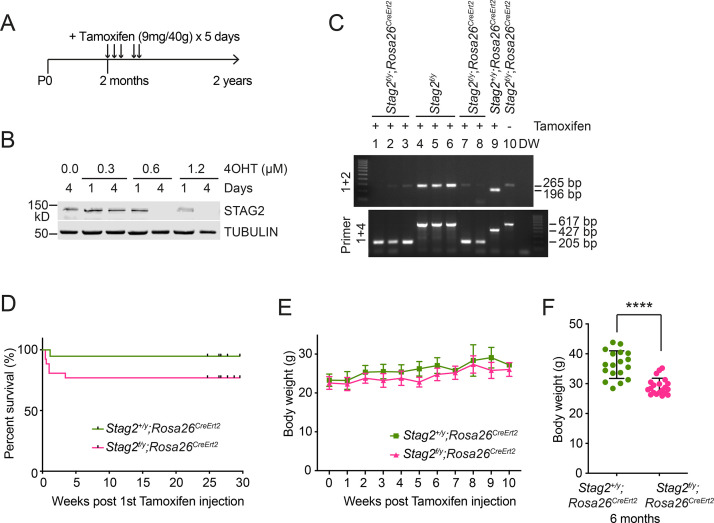
Generation of *Stag2* conditional knockout mice by gene targeting. (**A**) Experimental scheme of tamoxifen injection into adult *Stag2^f/y^;Rosa26^CreErt2^* and *Stag^+/y^;Rosa26^CreErt2^* mice. (**B**) Western blotting of cell extracts from *Stag2^f/y^;Rosa26^CreErt2^* mouse embryonic fibroblasts (MEFs) treated with or without 4-hydroxytamoxifen (4OHT). E12.5 MEFs were prepared from *Stag2^f/y^;Rosa26^CreErt2^* mouse embryos and subjected to 4OHT treatment. (**C**) PCR analysis of the genomic DNA extracted from the blood of indicated mice with the primers in Figure 1A. Only the floxed mice carrying *Rosa26^CreErt2^* (1,2,3,7,8) had their exon 8 excised in the condition of tamoxifen injection. (**D**) Survival curves of *Stag2^f/y^;Rosa26^CreErt2^* (*n* = 26) and *Stag^+/y^;Rosa26^CreErt2^* (*n* = 19) mice after tamoxifen injection. (**E**) Body weight of mice in (**D**). (**F**) Body weight of *Stag2^f/y^;Rosa26^CreErt2^* (*n* = 20) and *Stag^+/y^;Rosa26^CreErt2^* (*n* = 18) mice at 6 months post tamoxifen injection. ****p < 0.0001. Uncropped images of gels and blots in this figure are included in Figure 1—figure supplement 2—source data 1. Figure 1—figure supplement 2—source data 1.Uncropped images of gels and blots in Figure 1—figure supplement 2.

To study the functions of STAG2 in adult mice, we crossed the *Stag2^f/f^* mice with mice bearing the *Rosa26^CreErt2^* genomic insertion and generated *Stag2^f/y^;Rosa26^CreErt2^* progenies. The *Stag2^f/y^;Rosa26^CreErt2^* adult mice were injected with tamoxifen to induce *Stag2* deletion in the whole body (Figure 1—figure supplement 2A). Genotyping analysis of blood extracts showed that tamoxifen-induced efficient disruption of the *Stag2* gene locus in *Stag2^f/y^;Rosa26^CreErt2^* mice (Figure 1—figure supplement 2B, C). These *Stag2*-deficient adult mice did not show early onset of spontaneous tumor formation, indicating that *Stag2* mutation alone in somatic cells of mice is insufficient to induce tumorigenesis. The *Stag2*-deficient mice also did not have other obvious adverse phenotypes (Figure 1—figure supplement 2D), except that they had slightly lower body weight (Figure 1—figure supplement 2E, F), probably due to tissue homeostasis alterations reported by others (De Koninck et al., 2020).

STAG2 mutations are found in human cohesinopathy patients with mental retardation and neuropsychiatric behaviors (Soardi et al., 2017). To study the function of STAG2 in the nervous system, we generated *Stag2* conditional knockout mice (*Stag2* cKO) by crossing *Stag2^f/f^* mice with Nestin-Cre mice (Giusti et al., 2014; Figure 1C, D). The progenies were born in the Mendelian ratio, but *Stag2^f/y^;Nes^Cre^* pups presented growth retardation and premature death (Figure 1E, G). More than 50% *Stag2^f/y^;Nes^Cre^* mice died aged about 3 weeks while the rest died at about 4 months. *Stag2^f/y^* mice did not show differences discernible from wild-type (WT) littermates. Although *Stag2^f/y^;Nes^Cre^* mice did not present microcephaly, they exhibited frequent hydrocephaly that might contribute to their premature death. The *Stag2^f/y^;Nes^Cre^* mice displayed normal drinking and feeding behaviors (Figure 1H), but showed reduced plasma IGF-1 levels compared to the control mice (Figure 1J). *Stag2^f/y^;Nes^Cre^* mice showed forepaw and hindlimb clasping (Figure 1K) and limb tremors (Video 1), which were not seen in *Stag2^f/y^* mice. These data indicate that *Stag2* deficiency in the nervous system causes growth retardation and neurological defects.

**Video 1.** Neurological defects of brain-specific *Stag2* KO mice.

### *Stag2* ablation causes hypomyelination

**Figure 2.**
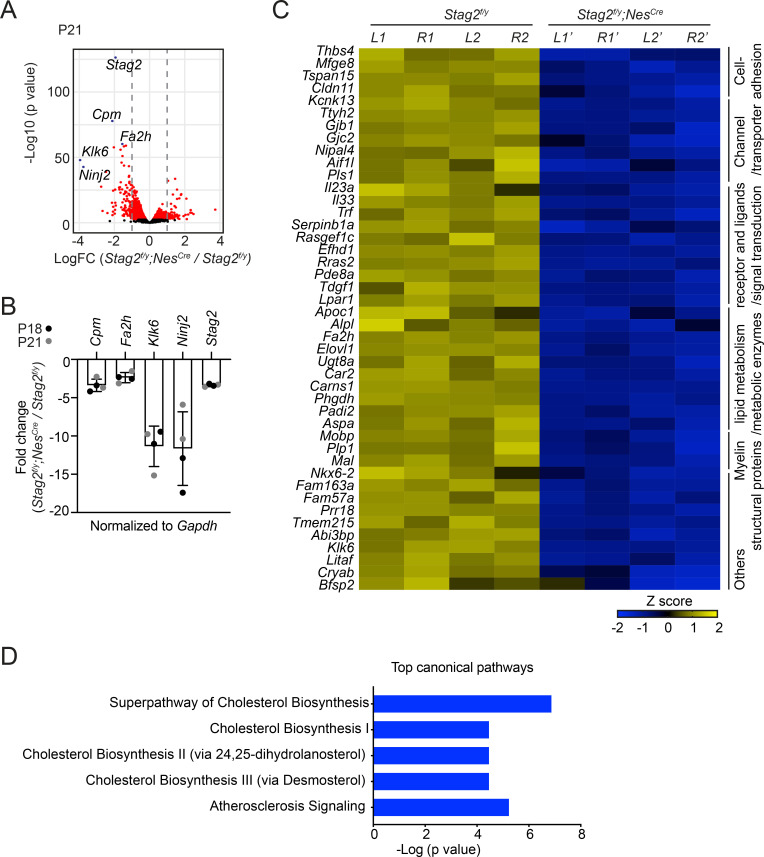
*Stag2* ablation in mouse brains downregulates the expression of myelin genes. (**A**) Volcano plot of bulk RNA-sequencing results of *Stag2^f/y^* and *Stag2^f/y^;Nes^Cre^* brain extracts. Top differentially expressed genes (DEGs) are colored blue and labeled. *n* = 4 pairs of P21 *Stag2^f/y^* and *Stag2^f/y^;Nes^Cre^* brain hemispheres were used for the comparison. (**B**) Reverse transcription quantitative PCR (RT-qPCR) analysis of the top downregulated genes in the brain extracts. *n* = 4 pairs of *Stag2^f/y^* and *Stag2^f/y^;Nes^Cre^* littermates were used. Mean ± standard deviation (SD). (**C**) Heatmap of the expression of myelin-enriched genes that were downregulated by more than twofolds in *Stag2^f/y^;Nes^Cre^* brains. *L1* and *R1*, left and right brain hemispheres of the *Stag2^f/y^#1* mouse. *L2* and *R2*, left and right brain hemispheres of the *Stag2^f/y^#2* mouse. *L1’* and *R1’*, left and right brain hemispheres of the *Stag2^f/y^;Nes^Cre^ #1* mouse. *L2’* and *R2’*, left and right brain hemispheres of the *Stag2^f/y^;Nes^Cre^#2* mouse. The biological pathways of these genes are labeled on the right. (**D**) The top 5 canonical pathways identified by ingenuity pathway analysis (IPA) of the DEGs. The complete gene list is used as the background.

Hematoxylin and eosin (H&E) staining of brain sections of *Stag2^f/y^;Nes^Cre^* mice did not reveal overt anatomical defects (Figure 2—figure supplement 1A). As revealed by immunohistochemistry assays using neuron- or astrocyte-specific antibodies, the differentiation of neurons and astrocytes in *Stag2*-deleted brains was largely normal (Figure 2—figure supplement 1B–E). To understand the origins of neurological defects caused by *Stag2* deletion, we analyzed the gene expression changes in *Stag2^f/y^;Nes^Cre^* mouse brains at post-natal day 21 by RNA-sequencing (RNA-seq) (Figure 2A). Compared with the control groups, 105 and 62 genes were significantly down- or upregulated by more than twofolds, respectively, in the *Stag2*-deficient brains. The decreased expression of top differentially expressed genes (DEGs) was confirmed by reverse transcription quantitative PCR (RT-qPCR) (Figure 2B). Among the 105 downregulated DEGs in the brains of *Stag2* cKO mice, 44 were enriched in myelin (Figure 2C; Thakurela et al., 2016). The ingenuity pathway analysis (IPA) pinpoints cholesterol biosynthesis pathways as the most affected canonical pathways (Figure 2D and Supplementary file 1). We further confirmed that the cholesterol biosynthesis precursors were reduced in *Stag2^f/y^;Nes^Cre^* brains (Figure 2—figure supplement 1F).

**Figure 2—figure supplement 1.**
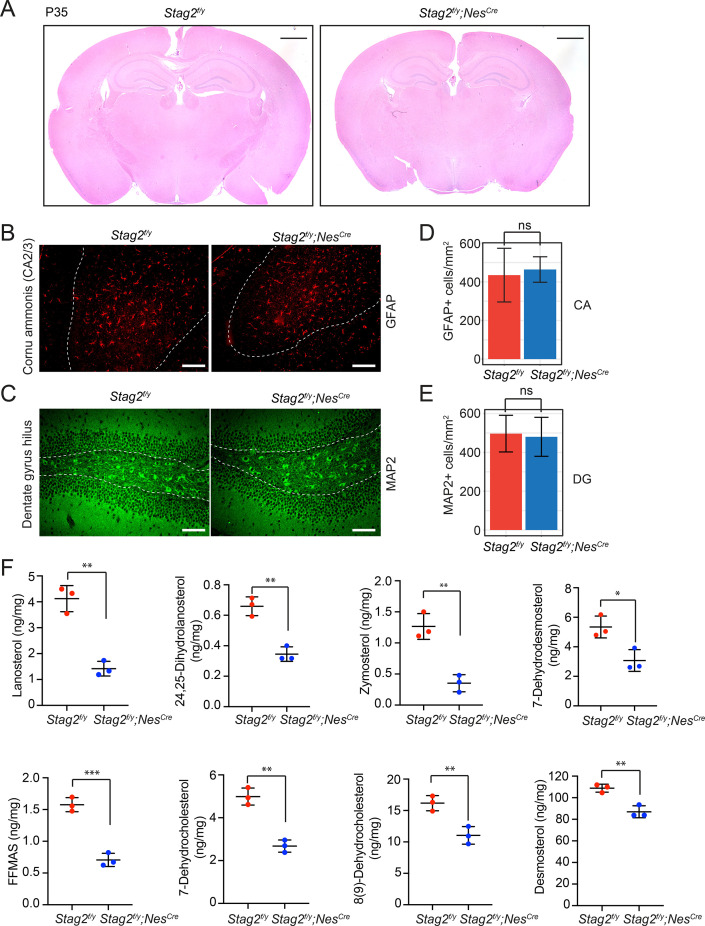
STAG2 deficiency in mouse brains attenuates cholesterol biosynthesis. (**A**) Hematoxylin and eosin (H&E) staining of the coronal sections of *Stag2^f/y^* and *Stag2^f/y^;Nes^Cre^* mouse brains. Scale bar = 1 mm. Immunohistochemistry staining of signature proteins of astrocytes (**B**) and neurons (**C**) on brain coronal sections of P18 or P21 *Stag2^f/y^* and *Stag2^f/y^;Nes^Cre^* mice. Scale bar = 100 μm. (**D**) Density of GFAP^+^ astrocytes in the cornu ammonis (CA) area (outlined with white dash lines in **B**) of the hippocampus. *n* = 3 mice per genotype. Mean ± SD; ns, not significant. (**E**) Density of MAP2^+^ neurons in the dentate gyrus hilus (outlined with white dash lines in **C**). n = 3 mice for *Stag2^f/y^* and n = 4 mice for *Stag2*^*f/y*^*;Nes*^*Cre*^. Mean ± SD; ns, not significant. (**F**) Mass spectrometry analysis of cholesterol precursors in *Stag2^f/y^* and *Stag2^f/y^;Nes^Cre^* brains. The cholesterol precursors mass measurement was normalized to the brain weight. *n* = 3 mice per genotype. *p < 0.05, **p < 0.01, ***p < 0.001; mean ± standard deviation (SD).

**Figure 2—figure supplement 2.**
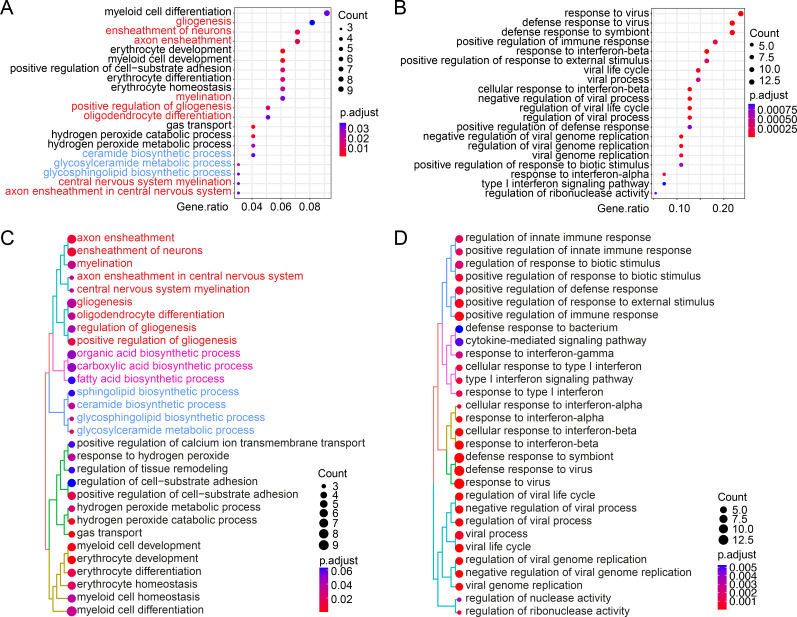
Over-representation analysis (ORA) of the RNA-sequencing (RNA-seq) results of the mouse brain samples. The enriched biological pathways identified by gene ontology of ClusterProfiler from the downregulated genes (**A**) or upregulated genes (**B**) with >twofold change between *Stag2^f/y^* and *Stag2^f/y^;Nes^Cre^* mice from the whole-brain RNA-seq dataset. Pathways of gliogenesis and myelination are highlighted in red. Pathways of membrane lipid biosynthesis are colored in blue. The top 20 pathways with the highest gene ratio are presented. Similarity tree plot of the top 30 enriched biological pathway as identified in A for the downregulated genes (**C**) or as in (**B**) for the upregulated genes (**D**). Pathways of myelination and gliogenesis are highlighted in red. Pathways of fatty acid biosynthesis and membrane lipid biosynthesis related to myelin sheath formation are colored in pink and blue, respectively.

Myelin is the membrane sheath that wraps around axons to facilitate rapid nerve conduction and maintain metabolic supply (Williamson and Lyons, 2018). Dynamic myelination in the central nervous system (CNS) is critical for proper neurodevelopment, and defective myelination is associated with autoimmune and neurodegenerative diseases (Mathys et al., 2019; Wolf et al., 2021). Cholesterol biosynthesis is essential for normal myelination (Hubler et al., 2018; Saher et al., 2005). Ensheathment of neurons and gliogenesis were among the top enriched biological pathways in downregulated DEGs (Figure 2—figure supplement 2). The innate immune response was among the top enriched pathways in the upregulated DEGs. We hypothesized that depletion of STAG2 caused myelination defects in the nervous system.

**Figure 3.**
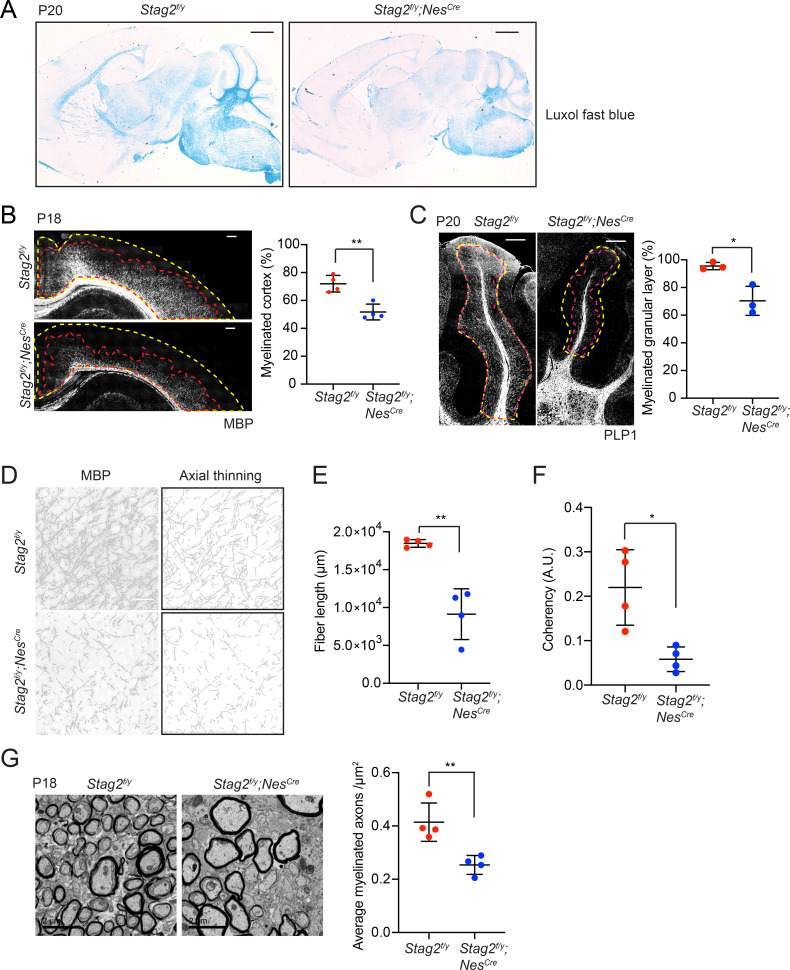
*Stag2* ablation in the nervous system compromises myelination during early postnatal development. (**A**) Luxol fast blue staining of the sagittal sections of *Stag2^f/y^* and *Stag2^f/y^;Nes^Cre^* brains. *n* = 3 animals per genotype. Scale bar = 1 mm. (**B**) Immunohistochemistry staining with the anti-MBP antibody in the cerebral cortex (left panel). Antibody-stained areas and DAPI staining regions are marked with red and yellow dashed lines, respectively. Scale bar = 200 μm. Quantification of the percentage of the myelinated cortex is shown in the right panel. *n* = 4 pairs of *Stag2^f/y^* and *Stag2^f/y^;Nes^Cre^* littermates were used (P18 or P21) for the comparison. **p < 0.01; mean ± standard deviation (SD). (**C**) Immunohistochemistry staining with the anti-PLP1 antibody in the cerebellum (left panel). Antibody-stained areas and DAPI staining regions are marked with red and yellow dashed lines, respectively. Scale bar = 200 μm. Quantification of the percentage of the myelinated cerebellum granular layer is shown in the right panel. *n* = 3 pairs of *Stag2^f/y^* and *Stag2^f/y^;Nes^Cre^* littermates were used (P20 or P25) for the comparison. *p < 0.05; mean ± SD. (**D**) Higher magnification images (left panel) of the immunohistochemistry staining with the anti-MBP antibody in (**B**). Images processed through axial thinning are shown in the right panel. Scale bar = 50 μm. Total fiber length (**E**) and fiber coherency (**F**) measured using the processed images in (**D**). *n* = 4 pairs of *Stag2^f/y^* and *Stag2^f/y^;Nes^Cre^* littermates were used (P18 or P21). *p < 0.05, **p < 0.01; mean ± SD. (**G**) Transmission electron microscopy images of the optic nerves (left panel). Scale bar = 2 μm. Quantification of myelinated axon distributions is shown in the right panel. *n* = 4 pairs of P18 *Stag2^f/y^* and *Stag2^f/y^;Nes^Cre^* littermates were used. *n* ≥ 10 fields of each mouse were taken, and the average distribution of myelinated axons were calculated for each mouse and plotted. **p < 0.01; mean ± SD.

Indeed, brain sections of *Stag2^f/y^;Nes^Cre^* mice showed greatly reduced luxol fast blue (LFB) staining compared to those of *Stag2^f/y^* and *Nes^Cre^* heterozygous mice at about P21 (Figure 3A and Figure 3—figure supplement 1A). Immunohistochemistry using antibodies against myelin proteins, Myelin basic protein (MBP) and Proteolipid protein 1 (PLP1), confirmed that *Stag2* cKO mice had significant defects in myelin fiber formation at P18-P21 (Figure 3B–F). In both cerebral cortex and cerebellum, there were fewer and sparser myelin fibers in *Stag2^f/y^;Nes^Cre^* mice, as compared to the *Stag2^f/y^* controls. Axon myelin ensheathment was further examined using transmission electron microscopy (Figure 3G). *Stag2^f/y^;Nes^Cre^* mice at P18 had significantly fewer myelin-wrapped axons at optic nerves. Collectively, these data indicate insufficient myelination in the *Stag2* cKO mice. Myelination predominantly occurs at 3 weeks after birth in the mouse. The timing of premature death of *Stag2* cKO mice is thus consistent with defective myelination as a contributing factor to the lethality.

**Figure 3—figure supplement 1.**
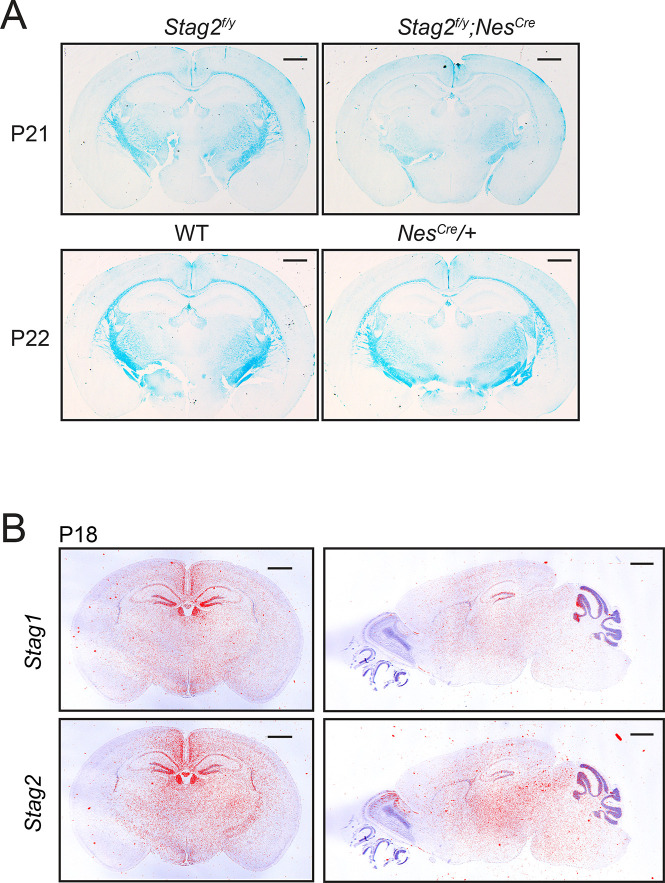
Brain-specific *Stag2* deletion impairs central nervous system (CNS) myelination. (**A**) Luxol fast blue staining of the coronal brain sections of mice with the indicated genotypes. *n* = 3 mice each for *Stag2^f/y^* and *Stag2^f/y^;Nes^Cre^* genotypes. *n* = 2 mice each for wild-type (WT) and *Nes^Cre^/+* groups. animals per genotype. Scale bar = 1 mm. (**B**) In situ hybridization of ^35^S-labeled RNA probes of the coronal (left) and sagittal (right) sections of WT mouse brains. Bright field images (purple) were overlaid with autoradiography images (red). Scale bar = 1 mm.

We examined *Stag1* and *Stag2* expression patterns in P18 WT mouse brains by in situ hybridization using isotope-labeled RNA probes (Figure 3—figure supplement 1B). Both *Stag1* and *Stag2* were expressed at high levels in hippocampus, medial habenula, neocortex, and cerebellum granular layer. Aside from these regions, the *Stag2* transcripts were detected at relatively high levels in subventricular zone, thalamus, fiber tracts, midbrain, and hindbrain regions. *Stag2* is thus ubiquitously expressed in the brain.

### STAG2 regulates transcription in OLs

**Figure 4.**
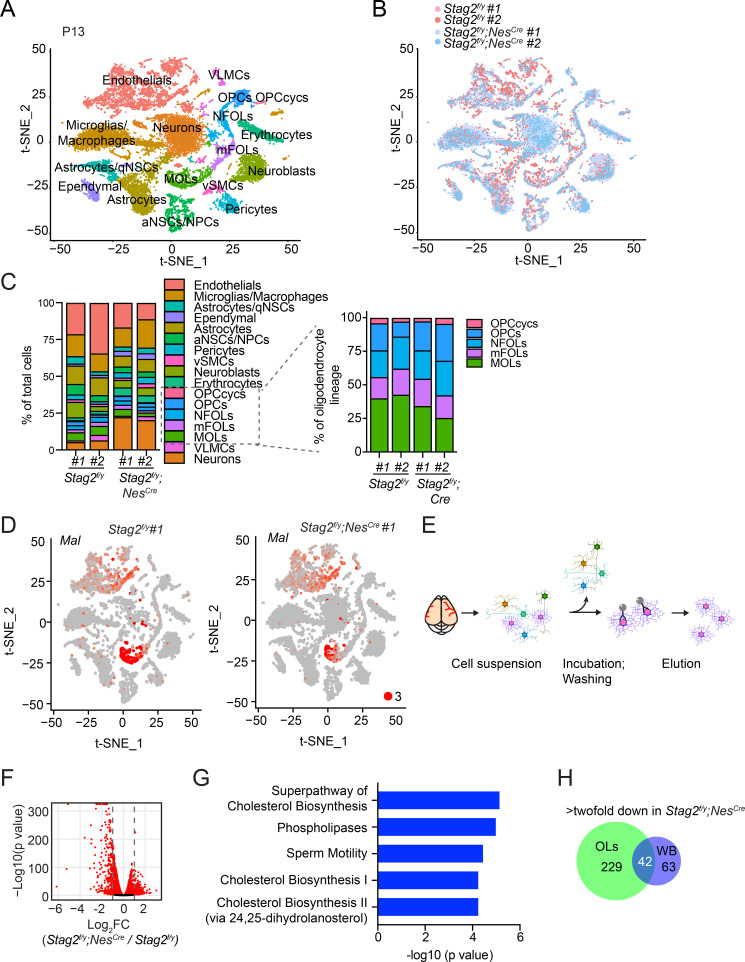
Deletion of *Stag2* in mouse brains causes differentiation delay and transcriptional changes in oligodendrocytes. (**A**) *t*-SNE plot of cell clusters in *Stag2^f/y^* and *Stag2^f/y^;Nes^Cre^* forebrains analyzed by single-cell RNA-sequencing (scRNA-seq). *n* = 2 mice of each genotype were used in the scRNA-seq analysis. aNSCs/NPCs, active neural stem cells or neural progenitor cells; Astrocytes/qNSCs, astrocytes or quiescent neural stem cells; OPCcycs, cycling oligodendrocyte (OL) progenitor cells; OPCs, OL progenitor cells; NFOLs, newly formed OLs; mFOLs, myelin-forming OLs; MOLs, matured OLs; VLMCs, vascular and leptomeningeal cells; vSMCs, vascular smooth muscle cells. (**B**) *t*-SNE clustering as in (**A**) but colored by genotype. (**C**) Left panel: cell-type composition and percentage as colored in (**A**). Right panel: percentage of cell clusters of the oligodendrocyte lineage. (**D**) FeaturePlot of a representative gene (*Mal*) specifically suppressed in MOLs of *Stag2^f/y^;Nes^Cre^* forebrains. A maximum cutoff of 3 was used. (**E**) Experimental scheme of the magnetic-activated cell sorting (MACS) of primary OLs. (**F**) Volcano plot of bulk RNA-sequencing (RNA-seq) results of *Stag2^f/y^* and *Stag2^f/y^;Nes^Cre^* primary OLs. (**G**) The top 5 canonical pathways identified by ingenuity pathway analysis (IPA) of the differentially expressed genes (DEGs) with more than twofold change in (**F**). The complete gene list is used as the background. (**H**) Commons DEGs shared between bulk RNA-seq analyses of the whole brains (WB) and primary OLs.

Oligodendrocytes (OLs) are responsible for myelination in the CNS. To examine whether the OL lineage was affected by *Stag2* deletion, we performed single-cell RNA-sequencing (scRNA-seq) analysis of *Stag2^f/y^;Nes^Cre^* and *Stag2^f/y^* forebrains at P13. As revealed by clusters in the t-SNE plot, the two genotype groups had similar cellular compositions (Figure 4A, B). All cell clusters were present in *Stag2^f/y^;Nes^Cre^* brains, again indicating generally normal neural cell differentiation. Cell-type identities were discovered with feature gene expression (Figure 4—figure supplement 1A). Based on the expression changes of *Stag2* and other cohesin genes in OLs, astrocytes, and neuronal lineages (Figure 4—figure supplement 1B–D), it is clear that *Stag2* ablation occurred early in the NPC stage and was maintained in all differentiated cell lineages.

**Figure 4—figure supplement 1.**
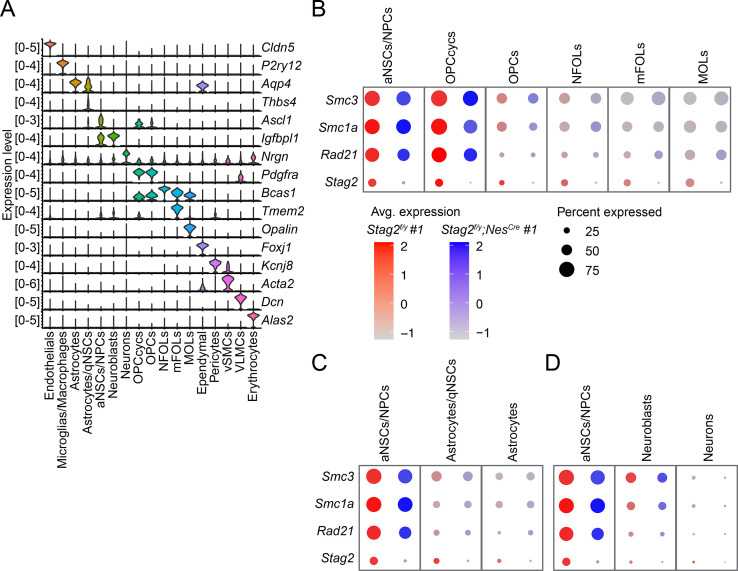
*Stag2* is ablated during early neural lineage differentiation of *Stag2* knockout mice. (**A**) Violin plot of the expression levels of feature genes of the indicated brain cell types. Dotplot showing the expression levels of cohesin subunit genes in the oligodendrocyte lineages (OLs; **B**), astrocytes (**C**), and neurons (**D**) and in the progenitor cells. *Stag2* expression is greatly diminished in the neuronal stem cells (NSCs) or neuronal progenitor cells (NPCs).

**Figure 4—figure supplement 2.**
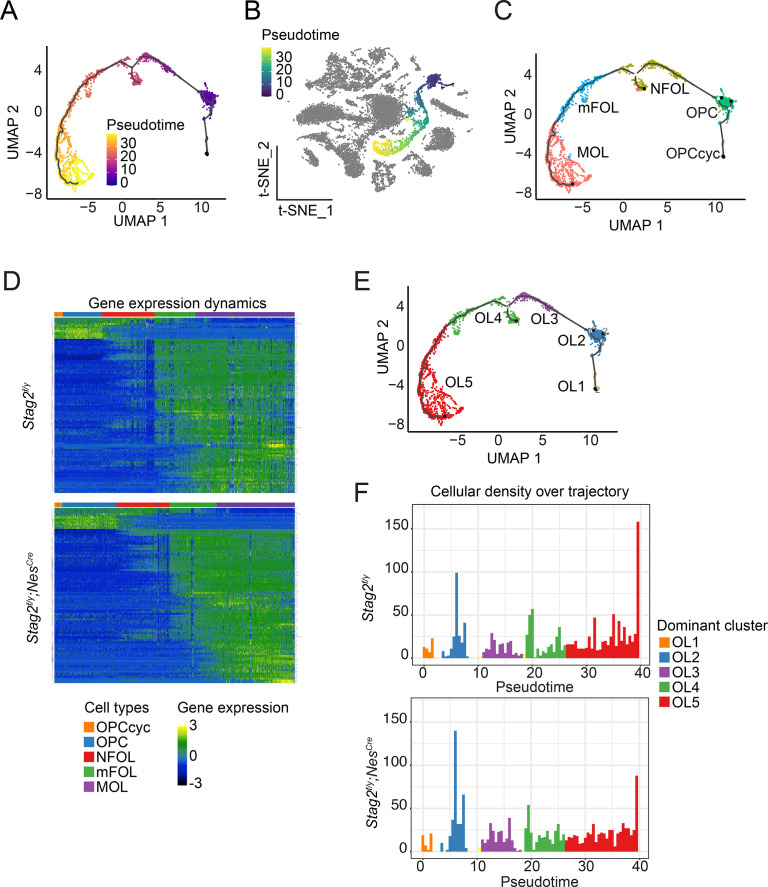
*Stag2* deletion causes differentiation delay in the oligodendrocyte lineage. (**A**) Trajectory inference analysis of oligodendrocyte (OL) lineage cells extracted from the single-cell RNA-sequencing (RNA-seq) dataset using Monocle3. Cells are colored from purple to yellow by pseudotime variables. (**B**) OL differentiation trajectory in the *t-SNE* plot. The OL lineage is colored from navy blue to yellow by pseudotime variables. Cells of other lineages are colored grey. (**C**) Distribution of the assigned OL cell types along the trajectory. (**D**) Heatmap of gene expression dynamics over pseudotime along the OL differentiation trajectory. Each row represents one of the top 100 most variable genes along pseudotime. Each column represents a single cell. (**E**) Reclustered OL subgroups in the trajectory inference analysis. (**F**) Cell density across pseudotime for the OL differentiation trajectory. Dominant clusters for each pseudotime bin are color labeled as in (**E**).

**Figure 4—figure supplement 3.**
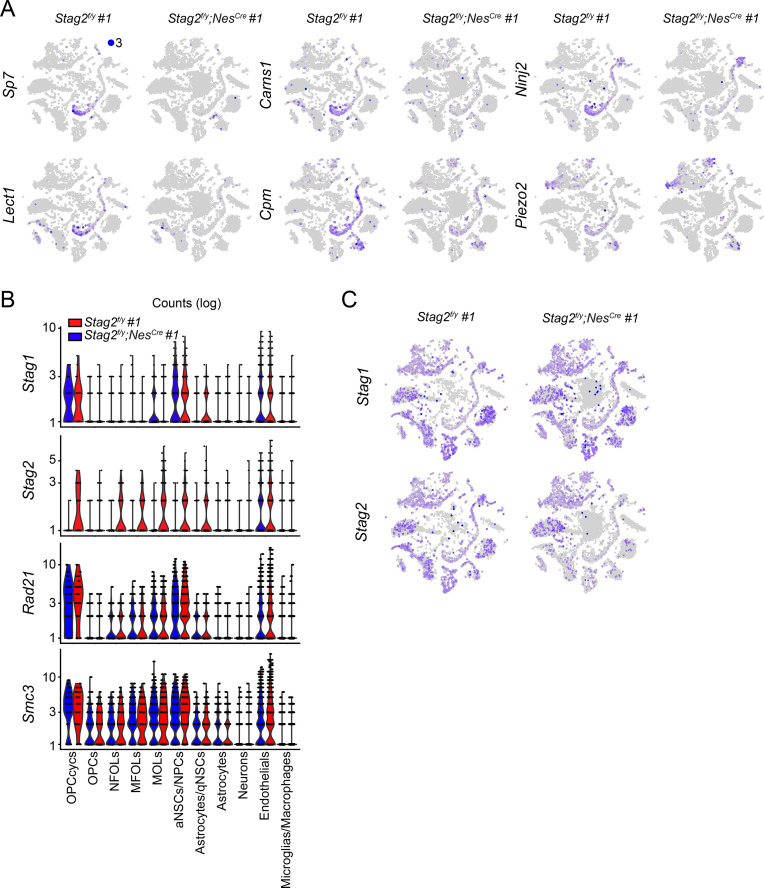
STAG2 regulates the transcription of oligodendrocyte genes. (**A**) FeaturePlot of the expression levels of representative downregulated genes in the *Stag2^f/y^;Nes^Cre^* whole brains. Maximum cutoff of 3 was used. (**B**) Violin plot of the expression of cohesin subunit genes in the indicated brain cell types from the single-cell RNA-sequencing (scRNA-seq) transcriptome analysis. (**C**) FeaturePlot of the expression of *Stag1* and *Stag2* in *Stag2^f/^*^y^ and *Stag2^f/y^;Nes^Cre^* forebrains. Maximum cutoff of 3 was used.

**Figure 4—figure supplement 4.**
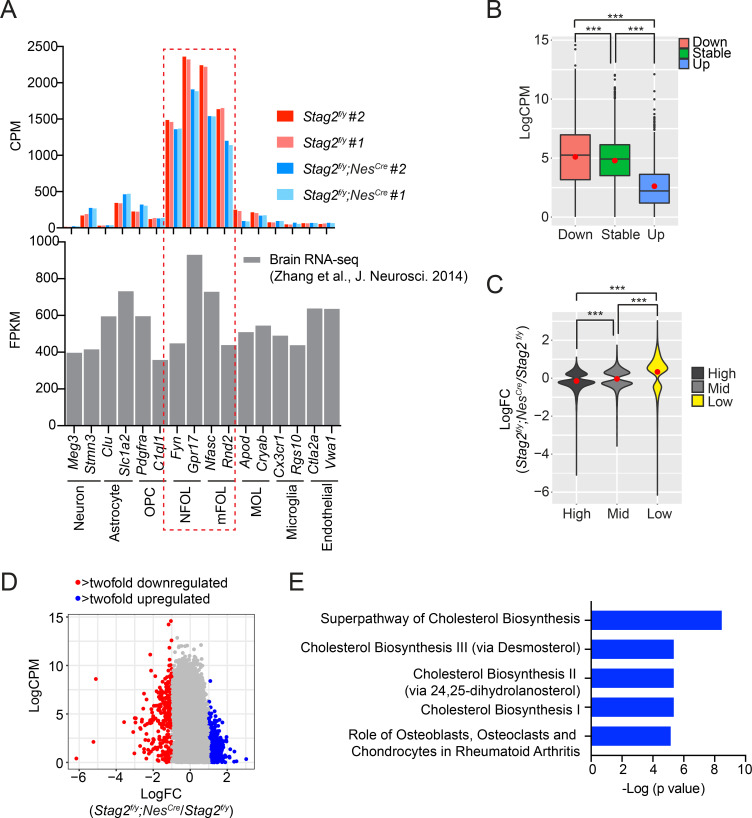
STAG2 regulates transcription in primary oligodendrocytes. (**A**) The expression levels of signature genes of indicated brain cell types in the isolated primary oligodendrocytes (OLs) in this study. The expression levels of the same set of signature genes in the individually isolated cell types from previous studies are shown below. NFOL and mFOL signature genes are highly enriched in the isolated primary OLs in this study. (**B**) Boxplot of the expression levels for genes in the indicated categories. Red dots represent the mean values. ***p < 0.001. Differentially expressed genes (DEGs) with more than 1.5-fold change are assigned as ‘down’ or ‘up’. Active genes with logFC between ±0.38 are assigned ‘stable’. (**C**) Violin plot of the expression changes for the active genes with different expression levels. Red dots represent the mean value. ***p < 0.001. (**D**) Scatter plot of the gene expression level against transcriptional changes. DEGs of the indicated categories are highlighted in red and blue.(**E**) Ingenuity pathway analysis (IPA) of the downregulated gene sets. The top 5 canonical pathways identified from IPA analysis of the downregulated genes in *Stag2*-deleted OLs. Downregulated genes with >twofold change were included in the analysis.

**Figure 4—figure supplement 5.**
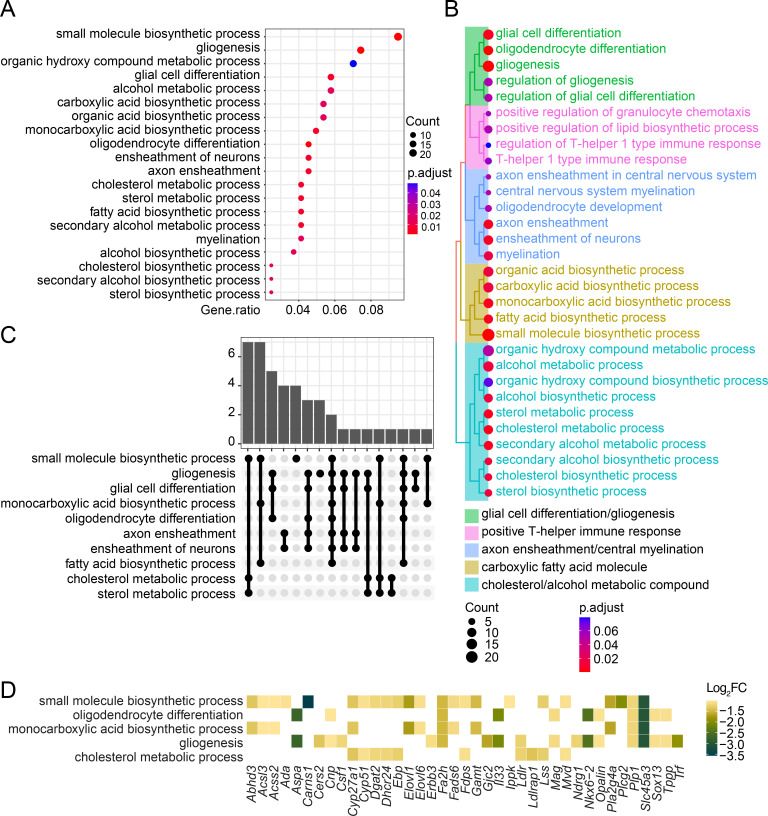
Over-representation analysis (ORA) of the downregulated genes in *Stag2*-deleted oligodendrocytes. (**A**) The enriched biological pathways identified from the downregulated genes with >twofold change in the *Stag2*-deleted primary oligodendrocytes. The top 20 pathways with the highest gene ratio are presented. (**B**) Treeplot of the top 30 enriched biological pathways identified as in (**A**). Pathways are grouped and colored by similarity. (**C**) Overlapped genes among the enriched biological pathways. Bar graph shows the number of overlapped genes among biological pathways. (**D**) Heatmap of the top enriched biological pathways and the expression change of related genes.

**Figure 4—figure supplement 6.**
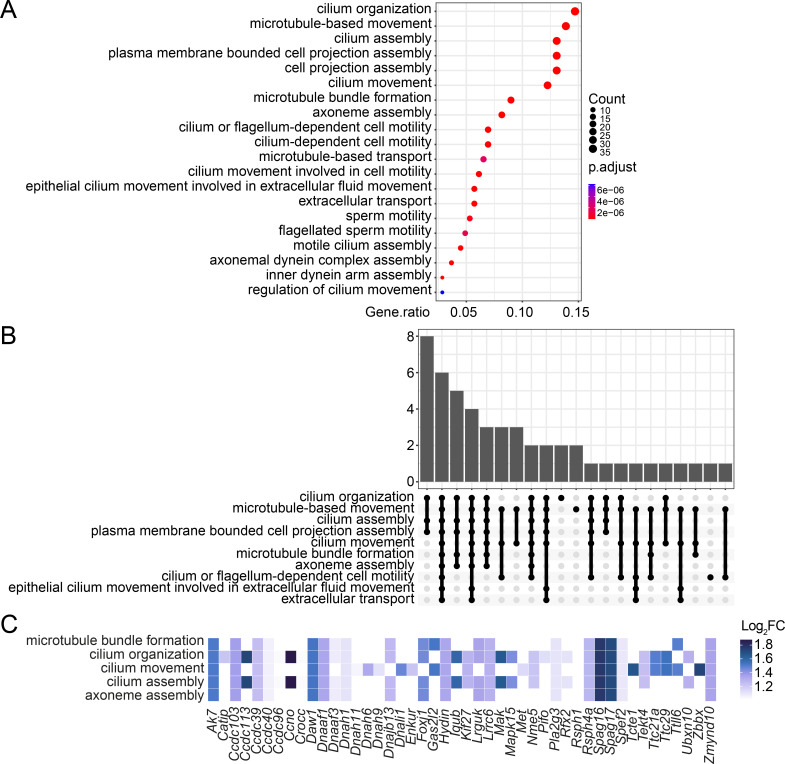
Over-representation analysis (ORA) of the upregulated genes in *Stag2*-deleted oligodendrocytes. (**A**) The enriched biological pathways identified from the upregulated genes with >twofold change in the *Stag2*-depleted primary oligodendrocytes. The top 20 pathways with the highest gene ratio are presented. (**B**) Overlapped genes among the enriched biological pathways. Bar graph shows the number of overlapped genes among biological pathways. (**C**) Heatmap of the top enriched biological pathways and the expression change of related genes.

The OL lineage consisted of five clusters: cycling OL progenitors (OPCcycs), OL progenitors (OPCs), newly formed OLs (NFOLs), myelin-forming OLs (mFOLs), and fully matured OLs (MOLs). Quantification of the distributions of these five cell types within the OL lineage revealed a mild reduction in the proportion of MOLs in *Stag2^f/y^* forebrains (Figure 4C). We noticed that a higher percentage of neurons was recovered in the *Stag2^f/y^;Nes^Cre^* group. Since the bulk RNA-seq results did not show global upregulation of neuron-specific genes, we suspect that neurons in *Stag2^f/y^;Nes^Cre^* had fewer myelin-wrapped axons and were easier to be dissociated and kept alive during our library preparation for scRNA-seq. Thus, from the transcriptome analysis, we did not observe overt defects in most neural cell differentiation in the *Stag2*-deficient forebrain regions.

We then performed trajectory inference and pseudotime analysis of the OL lineage (Figure 4—figure supplement 2A, B). Consistent with our cell-type assignment, pseudotime variables indicated continuous differentiation from OPCs to NFOLs, mFOLs, and MOLs (Figure 4—figure supplement 2C,D). The reclustering of single cells in the OL lineage along the pseudotime path revealed that more cells were present in the terminal maturation stages in the *Stag2^f/y^* brains (Figure 4—figure supplement 2E, F). Conversely, more cells were retained at the undifferentiated stages in the *Stag2^f/y^;Nes^Cre^* brains. Strikingly, some myelination genes, including Myelin and lymphocyte protein (*Mal)*, were specifically repressed in *Stag2^f/y^;Nes^Cre^* MOLs, with their expression in nonneural cells unaltered (Figure 4D and Figure 4—figure supplement 3A). These observations suggest that STAG2 deficiency delays the maturation of OLs and compromises myelination-specific gene expression in mature OLs. Interestingly, compared to *Stag2* and genes encoding other cohesin core subunits, *Stag1* transcripts are less abundant in the OL lineage, except for cycling OPCs (Figure 4—figure supplement 3B, C). The low expression of *Stag1* in mature OLs might make these cells more dependent on *Stag2* for function.

To confirm the transcriptional defects in the OL lineage caused by *Stag2* deletion, we isolated primary OLs at intermediate differentiation stages from *Stag2^f/y^;Nes^Cre^* and *Stag2^f/y^* forebrains at P12-P14 with antibody-conjugated magnetic beads and conducted bulk RNA-seq analysis (Figure 4E). For both genotypes, the marker genes for NFOL and mFOLs were highly expressed in the isolated primary OLs (Figure 4—figure supplement 4A), suggesting that they mainly contained these two cell types. In *Stag2*-deleted OLs, 271 and 292 genes were downregulated or upregulated by more than two folds, respectively (Figure 4F and Supplementary file 2). Intriguingly, the downregulated genes were generally highly expressed in WT cells, whereas the upregulated genes had low expression levels in WT cells (Figure 4—figure supplement 4B–D). The top pathways enriched in the downregulated DEGs included the cholesterol and small molecule biosynthetic pathways and oligodendrocyte differentiation (Figure 4G and Figure 4—figure supplement 5). Cilium organization and assembly are the top enriched pathways in the upregulated DEGs (Figure 4—figure supplement 6). Among the 105 downregulated DEGs identified by RNA-seq analysis of the whole brain of *Stag2*-deficient mice, 42 were also differentially expressed in primary oligodendrocytes (Figure 4H). The cholesterol biosynthetic pathways were recognized as the major altered pathways (Figure 4—figure supplement 4E). Thus, defective cholesterol biosynthesis and oligodendrocyte differentiation likely underly hypomyelination and neurological defects in *Stag2* cKO mice.

**Figure 5.**
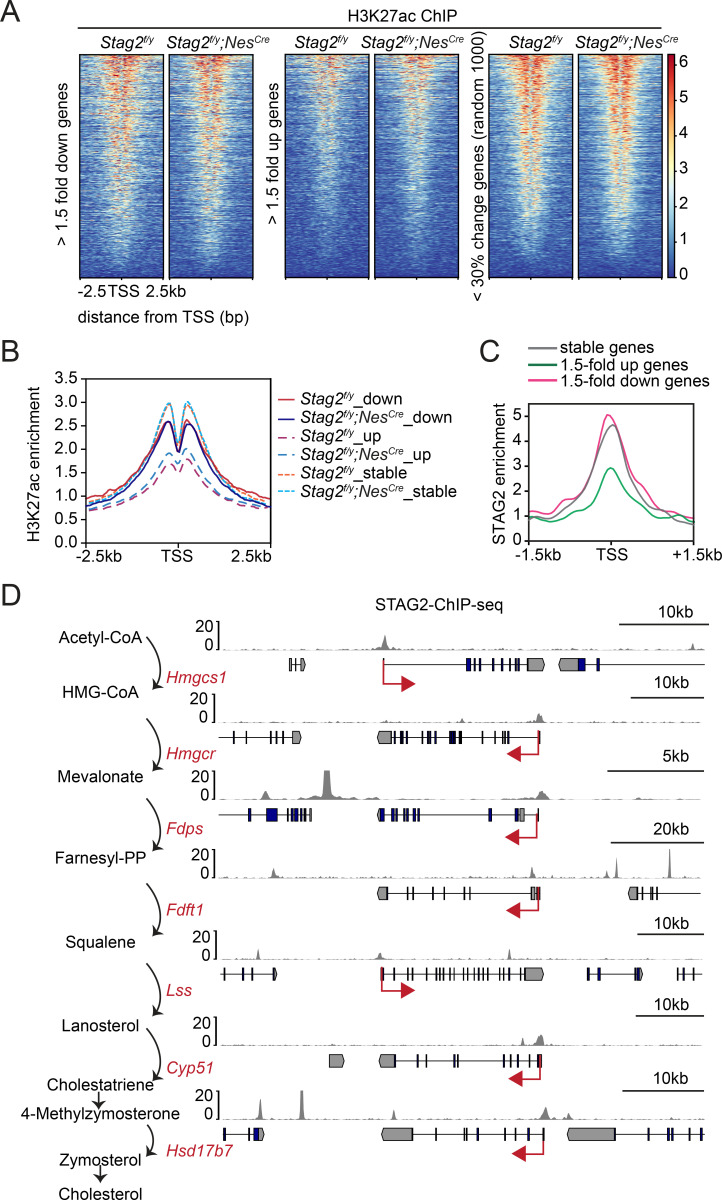
Enrichment of STAG2 and histone modifications at gene promoters. (**A**) Heatmap of H3K27ac chromatin immunoprecipitation sequencing (ChIP-seq) signal enrichment in the promoter regions of genes in the indicated categories. (**B**) Density profile of H3K27ac ChIP-seq signal enrichment in the promoter regions of genes in the indicated categories as in (**A**). (**C**) Density profile of STAG2 ChIP-seq signal enrichment in the promoter regions of genes in the indicated categories as in (**A**). (**D**) Binding of STAG2 at the genomic loci of downregulated genes that encode cholesterol biosynthetic enzymes as revealed by ChIP-seq.

We performed chromatin immunoprecipitation sequencing (ChIP-seq) experiments to examine the enrichment of the active transcription mark H3K27ac in *Stag2^f/y^* and *Stag2^f/y^;Nes^Cre^* OLs and found that *Stag2* loss did not appreciably affect H3K27Ac enrichment at transcription start sites (TSSs) (Figure 5A, B). Consistent with our RNA-seq results, the upregulated genes had much lower H3K27ac enrichment near their TSS, indicating that they were less active. We then checked the genomic distribution of STAG2 by ChIP-seq. Among other genomic loci, STAG2 was enriched at TSS of stable and downregulated genes, including genes in the cholesterol biosynthesis and myelination pathways (Figure 5C, D, Figure 5—figure supplement 1, and Supplementary file 2). Among the 271 downregulated DEGs, there were 210 genes (77%) with STAG2 enrichment near the transcriptional start site (TSS ± 2 kb). Thus, STAG2 occupied the promoter regions of many downregulated DEGs in oligodendrocytes. It was enriched at the TSS of upregulated genes to a lesser extent, with only 117 of the 292 (40%) upregulated DEGs exhibiting STAG2 ChIP-seq peaks at their TSS ± 2 kb regions. *Stag2* loss might have indirectly affected the expression of these less active genes.

**Figure 5—figure supplement 1.**
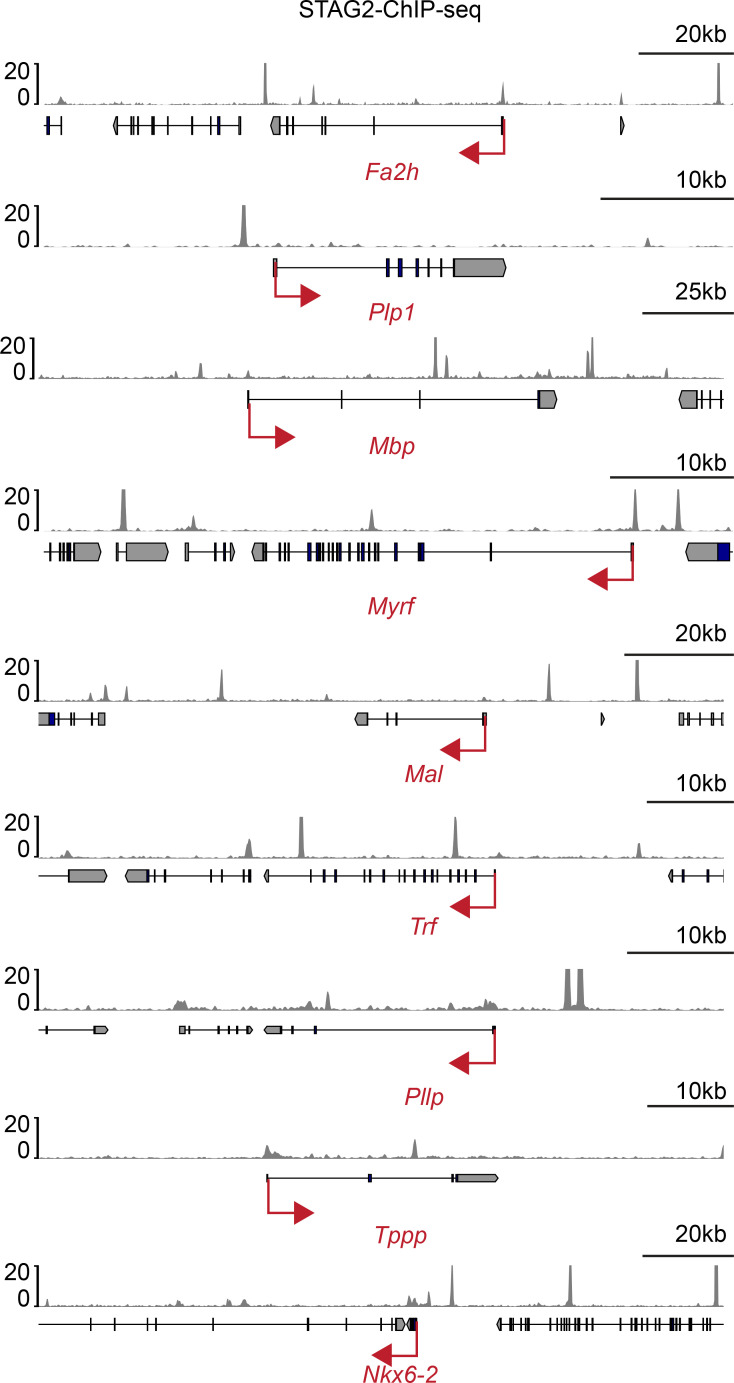
STAG2 occupies the promoters of myelination genes. Tracks of STAG2 binding at the genomic loci of the downregulated myelination genes. Red arrows indicate the transcription direction.

### *Stag2* deletion does not alter compartments or TADs in OLs

**Figure 6.**
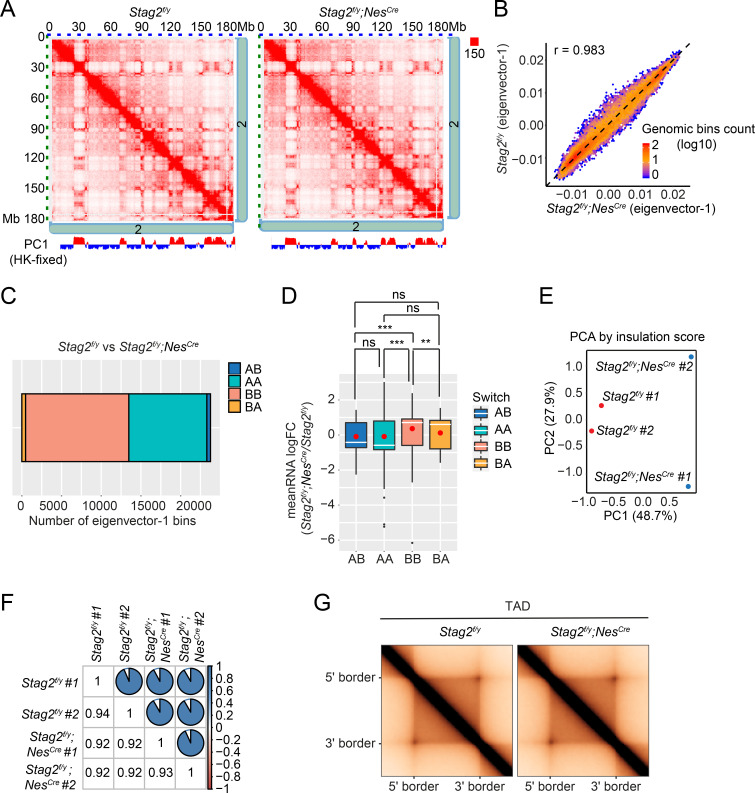
Loss of *Stag2* does not alter compartments and topologically associated domains (TADs) in oligodendrocytes. (**A**) Representative snapshots of balanced Hi-C contact matrices of chromosome 2. Tracks of eigenvector-1 fixed with housekeeping genes are shown below, with A and B compartments shown in red and blue, respectively. (**B**) Hexbin plot of eigenvector-1 for genomic bins (100 kb) in *Stag2^f/y^* and *Stag2^f/y^;Nes^Cre^* oligodendrocytes (OLs). (**C**) Chromatin bins were classified into four categories based on the eigenvector sign and whether it has switched with a delta bigger than 1.5. AB, changing from compartment A in *Stag2^f/y^* to compartment B in *Stag2^f/y^;Nes^Cre^*; BA, from B in *Stag2^f/y^* to A in *Stag2^f/y^;Nes^Cre^*; AA, A in both *Stag2^f/y^* and *Stag2^f/y^;Nes^Cre^*; BB, B in both *Stag2^f/y^* and *Stag2^f/y^;Nes^Cre^*. (**D**) Boxplot of averaged gene expression change of differentially expressed genes (DEGs) (RNA logFC cutoff of ±0.58) inside each genomic bin. Bins counted: AA, 1646; AB, 56; BA, 69; BB, 910. Red dots represent the mean value. An unpaired Wilcoxon test was used for the statistical analysis. **p < 0.01; ***p < 0.001; ns, not significant. Principal component (**E**) and similarity (**F**) analysis performed using the insulation score at 10 kb resolution. (**G**) Aggregate TAD analysis on the 10 kb merged Hi-C matrices using TADs called from the merged samples of *Stag2^f/y^* at 10 kb resolution.

To investigate whether chromosome conformation was altered by *Stag2* deletion and whether that caused transcription dysregulation, we performed high-dimensional chromosome conformational capture (Hi-C) analysis of primary OLs isolated from *Stag2^f/y^* and *Stag2^f/y^;Nes^Cre^* mice in biological replicates (Figure 6 and Figure 6—figure supplement 1). We observed few compartment switching events in *Stag2*-deleted cells (Figure 6A–C). Virtually all genomic regions in *Stag2*-deleted cells were kept in their original compartment categories (AA or BB) (Figure 6C). Only a very small number of genomic regions switched compartments (AB or BA). Consistent with the RNA-seq data, analysis of average gene expression changes of DEGs in these genomic regions revealed that more genes located in the transcriptionally active A compartment (AA) were repressed in *Stag2*-deleted cells and more genes in the transcriptionally silent B compartment (BB) were upregulated (Figure 6D and Figure 6—figure supplement 1C). Genes that switched from the A compartment to the B compartment were not more repressed compared to those that remained in the A compartment. Likewise, compared to genes that stayed in the B compartment, genes located in chromatin regions that switched from compartment B to A were not significantly activated. Acute depletion of all forms of cohesin eliminates TAD formation (Wutz et al., 2017). In contrast, deletion of *Stag2* had minimal impact on TAD formation in oligodendrocytes (Figure 6E–G and Figure 6—figure supplement 1D), suggesting that STAG1-cohesin compensates for the loss of STAG2-cohesin in spatial organization of chromatin at larger than megabase scales. Therefore, our analyses did not uncover evidence for compartment switching and TAD alterations being the underlying cause for the observed gene expression changes in STAG2-deficient OLs.

**Figure 6—figure supplement 1.**
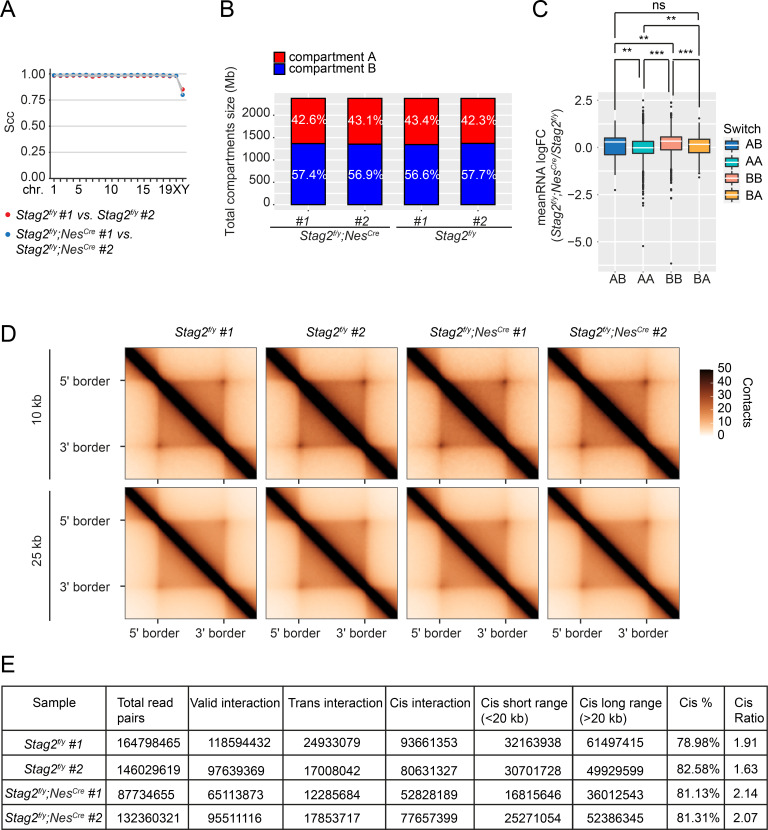
*Stag2*-deleted OLs do not present significant changes in compartments and topologically associated domains (TADs). (**A**) HiCRep analysis for reproducibility of the Hi-C replicates. The stratum-adjusted correlation coefficient (SCC) is calculated for each pair of duplicates for all chromosomes at 25 kb resolution. (**B**) Compartment compositions of the indicated samples. (**C**) Boxplot of the average gene expression change for all the differentially expressed genes (false-discovery rate [FDR] <0.05) inside each genomic bin. Bins counted: AA, 5806; AB, 155; BA, 251; BB, 2659. The unpaired Wilcoxon test was used for the statistical analysis. **p < 0.01; ***p < 0.001; ns, not significant. (**D**) Aggregate TAD analysis on the replicates of 10 kb Hi-C matrices using TADs called from the merged samples of *Stag2^f/y^* at 10 or 25 kb resolution. (**E**) Hi-C sample statistics of total read pairs, valid pairs, and cis-pairs using HiC-Pro.

### Promoter-anchored loops were reduced in *Stag2*-deleted OLs

**Figure 7.**
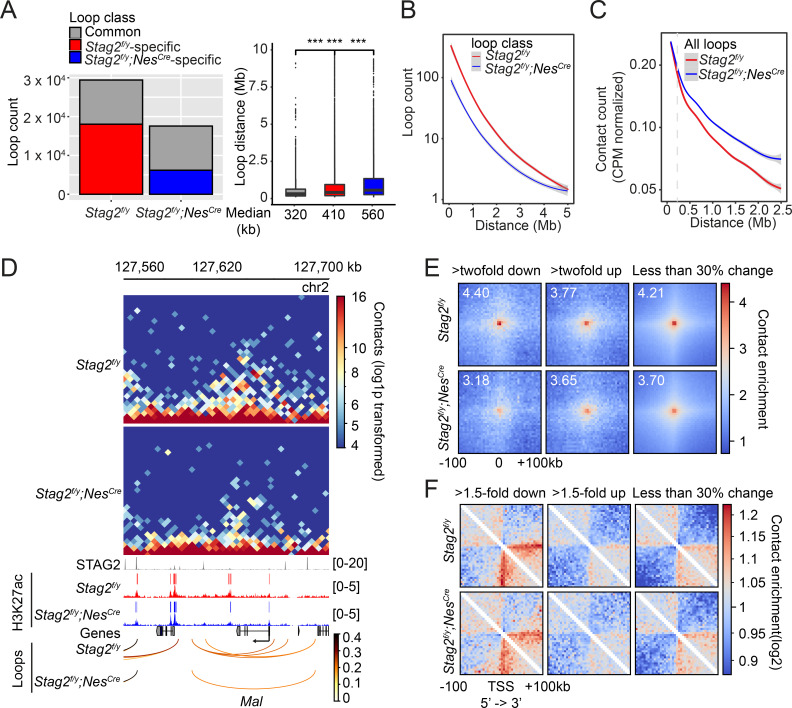
*Stag2* deletion impairs the formation of total and promoter-anchored loops in oligodendrocytes. (**A**) Loop counts (left panel) and length (right panel) in the indicated categories of *Stag2^f/y^* and *Stag2^f/y^;Nes^Cre^* oligodendrocytes (OLs). ***p < 0.001. (**B**) Loop counts plotted against loop length (from 0 to 5 Mb) of *Stag2^f/y^* and *Stag2^f/y^;Nes^Cre^* OLs. (**C**) Normalized contact counts for loops across different genomic distances in *Stag2^f/y^* and *Stag2^f/y^;Nes^Cre^* OLs. (**D**) Representative snapshots of contact maps at the *Mal* gene locus.hic files generated by HiC-Pro were converted to.cool format for plotting at 5 kb resolution. Tracks and narrow peaks from STAG2 and H3K27ac chromatin immunoprecipitation sequencing (ChIP-seq) as well as the loops are plotted below. Transcription direction is indicated by the black arrow. (**E**) Pile-up analysis of loop ‘dots’-centered local maps for the promoter-anchored loops of genes in the indicated categories. The maps are balanced, normalized by distance, and plotted at 5 kb resolution. The numbers indicate the enrichment of the central pixel over the upper left and bottom right corners. (**F**) Pile-up analysis of the local contact maps centered around the transcription start site (TSS) of genes in the indicated categories. Transcription directions are indicated below. 1000 stable genes are chosen randomly and used for the analysis. The maps are balanced, normalized by distance, and plotted at 5 kb resolution. Diagonal pixels are omitted.

While TAD boundaries are largely conserved among species and cell types, chromatin interactions within each TAD are more flexible and variable in cells undergoing differentiation, tumorigenesis, and reprogramming (Dixon et al., 2015; Dixon et al., 2012). Among the intra-TAD chromatin interactions, the enhancer–promoter loops are particularly important for transcription and are often cell-type specific. We examined whether chromatin loops in OLs were affected by *Stag2* loss. Compared to *Stag2^f/y^* OLs, *Stag2^f/y^;Nes^Cre^* OLs had significantly fewer loops across almost all genomic distances (Figure 7A, B and Figure 7—figure supplement 1). The common and genotype-specific loops are reproducible in each replicate. Loops specific to *Stag2^f/y^;Nes^Cre^* OLs, which were likely mediated by STAG1-cohesin, were longer than STAG2-dependent *Stag2^f/y^*-specific loops. When genomic distances exceeded 0.25 Mb, the loops from *Stag2^f/y^;Nes^Cre^* cells gradually gained higher scores over loops from *Stag2^f/y^* cells (Figure 7C). Therefore, STAG1-cohesin cannot completely compensate for STAG2-cohesin during loop formation. STAG1-cohesin-mediated loops are relatively longer than STAG2-cohesin-mediated loops, consistent with published findings in HeLa cells (Wutz et al., 2020).

**Figure 7—figure supplement 1.**
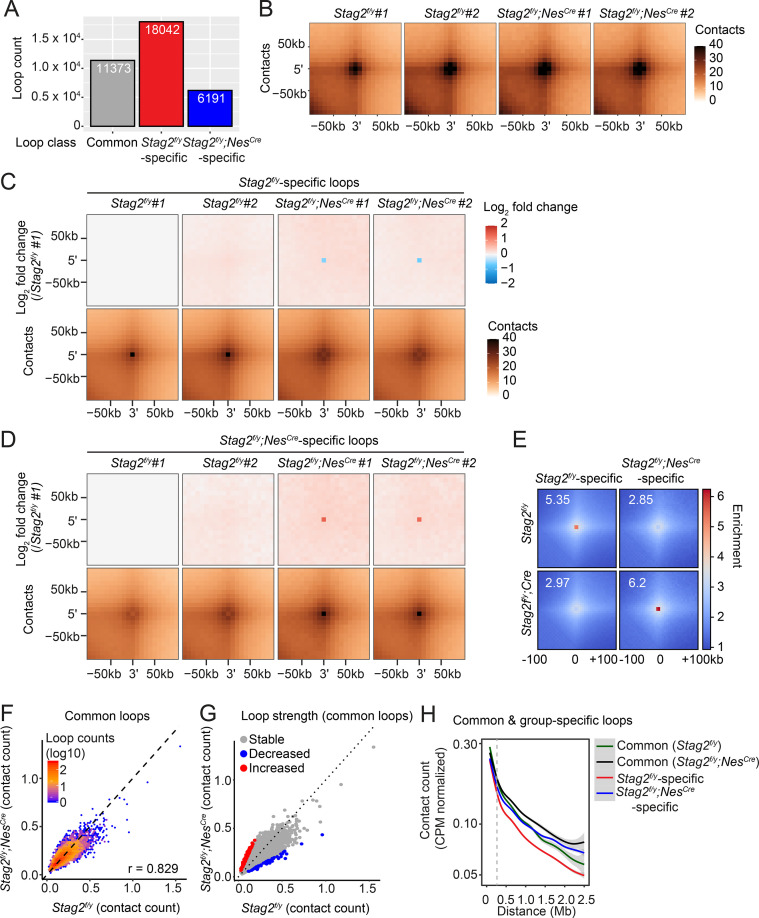
*Stag2* deletion reduces chromatin loops in oligodendrocytes. (**A**) Loop numbers of the indicated categories. (**B**) Aggregate peak analysis on the replicates of 10 kb Hi-C matrices using loops called from the merged samples of *Stag2^f/y^* or *Stag2^f/y^;Nes^Cre^* mice. Aggregate peak analysis performed on the replicates of 10 kb Hi-C matrices using the group-specific loops. *Stag2^f/y^*-specific loops are used in (**C**) and *Stag2^f/y^;Nes^Cre^*-specific loops are used in (**D**). The log_2_ fold change over *Stag2^f/y^ #1* is plotted on the top panels. (**E**) Pile-up analysis of loop ‘dots’-centered local contact maps for loops specific to *Stag2^f/y^* or *Stag2^f/y^;Nes^Cre^* oligodendrocytes (OLs). (**F**) Hexbin plot of contact counts of common loops in *Stag2^f/y^* and *Stag2^f/y^;Nes^Cre^* OLs. (**G**) Scatter plot of contact counts of common loops in *Stag2^f/y^* and *Stag2^f/y^;Nes^Cre^* OLs. Loops with significantly changed strength in *Stag2^f/y^;Nes^Cre^* OLs are highlighted in red (increased) and blue (decreased). Log_2_FC threshold of 1 was used. (**H**) Normalized contact counts for loops in the indicated categories across different genomic distances.

**Figure 7—figure supplement 2.**
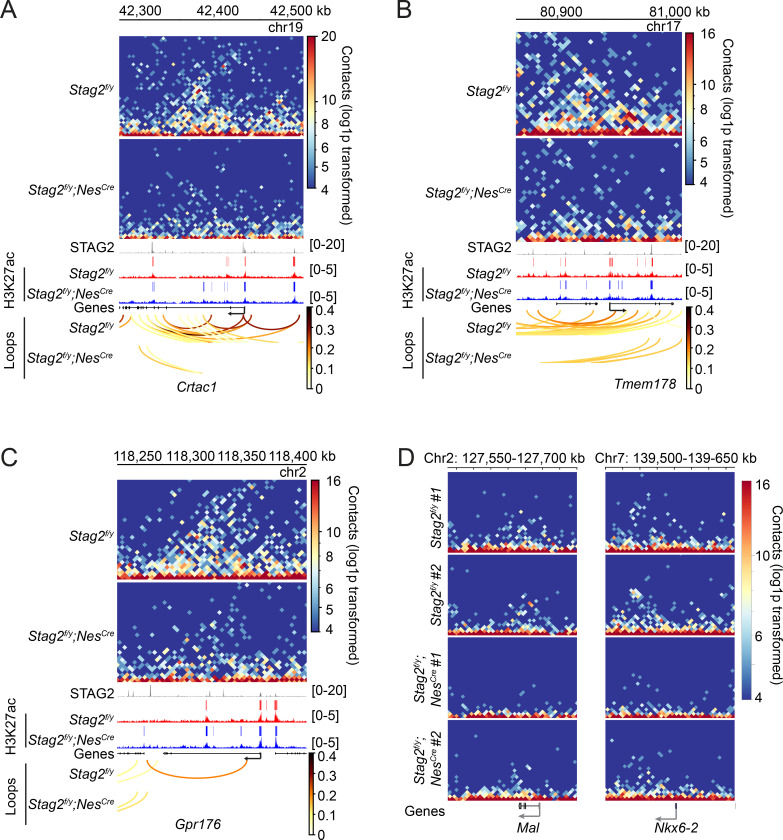
STAG2 controls local chromatin looping at differentially expressed genes. (**A–C**) Snapshots of the contact maps at the indicated differentially expressed genes. Tracks and peaks from STAG2 and H3K27ac chromatin immunoprecipitation sequencing (ChIP-seq) as well as loops are shown below. Transcription directions are indicated by arrows. (**D**) Representative snapshots of contact maps of the replicate samples at *Mal* and *Nkx6-2* genomic loci.hic files generated by HiC-Pro were converted into.cool format. Graphs were plotted with pyGenomeTracks at 5 kb resolution. Transcription orientations are indicated with the black arrows.

**Figure 7—figure supplement 3.**
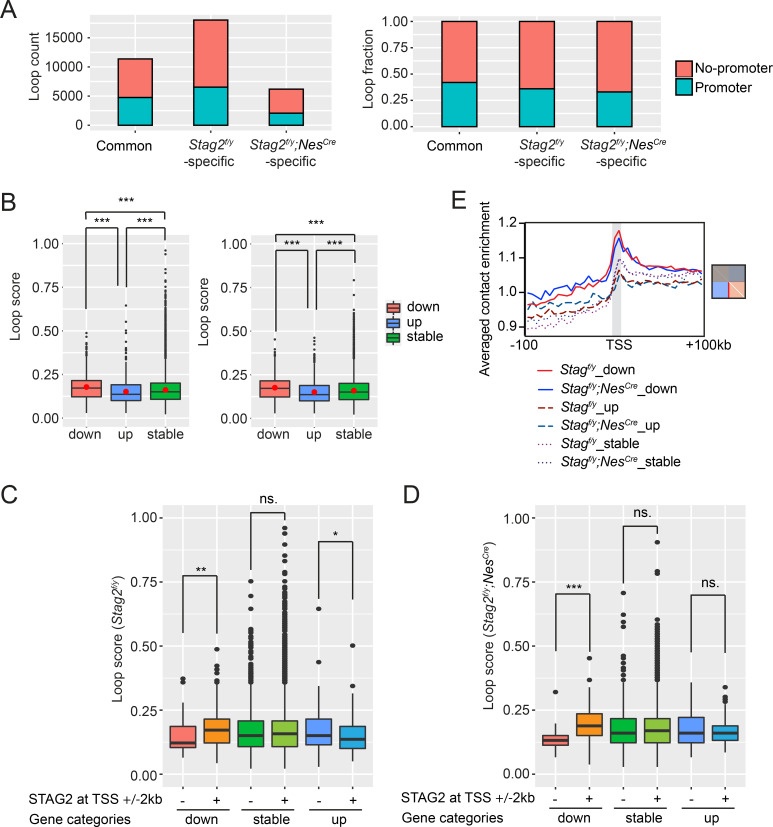
STAG2 regulates the formation of promoter-anchored loops in oligodendrocytes. (**A**) Loop counts (left panel) and fractions (right panel) of loops anchored at promoter or nonpromoter regions in the indicated categories. (**B**) Loop score of the loops in Figure 7E. Loop score from *Stag2^f/y^* oligodendrocytes (OLs) was used for common loops on the left, and loop score from *Stag2^f/y^;Nes^Cre^* OLs was used for common loops on the right. The unpaired Wilcoxon test was used for the statistical analysis. ***p < 0.001. (**C, D**) Loop score of loops anchored at differentially expressed genes (DEGs) and stable gene promoters with or without STAG2 enrichment. Loops from *Stag2^f/y^* OLs are used for the analysis in (**C**) and loops from *Stag2^f/y^;Nes^Cre^* OLs are used for the analysis in (**D**). Unpaired Wilcoxon test was used for the statistical analysis. *p < 0.05; **p < 0.01; ***p < 0.001; ns, not significant. (**E**) Profile plot of the average enrichment score for the bottom half of each graph panel in Figure 7F. Diagonal pixels were omitted.

We then tested whether the loop number decrease in *Stag2*-deficient cells could be a cause for transcriptional changes. When examining the local Hi-C maps, we noticed that loops anchored at gene promoters, including those of downregulated genes, were reduced in *Stag2^f/y^;Nes^Cre^* oligodendrocytes (Figure 7D, Figure 7—figure supplement 2A–C, and Supplementary file 2). The effects were again reproducible in each replicate (Figure 7—figure supplement 2D). Promoter-anchored loops (P-loops) can potentially be promoter–promoter links, promoter–enhancer links, and gene loops. The total number of P-loops was proportionally decreased in *Stag2^f/y^;Nes^Cre^* cells (Figure 7—figure supplement 3A). Moreover, the loops anchored at the downregulated genes were stronger than those at upregulated and stable genes (Figure 7—figure supplement 3B). We then compared P-loops associated with DEGs using pile-up analysis of local contact maps. Loop enrichment at promoters of downregulated genes was reduced in *Stag2^f/y^;Nes^Cre^* cells to a greater extent than that at promoters of upregulated and stable genes (Figure 7E). Among the 162 downregulated DEGs with reduced promoter-anchored loops in the *Stag2*-depleted cells, 137 genes (85%) had STAG2 peaks in their promoter regions (TSS ± 2 kb). The loops anchored at downregulated genes with STAG2 binding had significantly higher loop scores, compared to those with no STAG2 binding (Figure 7—figure supplement 3C, D). This difference was still observed in *Stag2*-deleted cells, suggesting that the stronger looping at these gene promoters might be maintained by STAG1-cohesin or other factors in these cells. By contrast, the loops anchored at upregulated genes with STAG2 binding had lower loop scores. These differences became insignificant in the *Stag2*-deleted cells. The loop scores of loops anchored at stable genes were not affected by STAG2 occupancy. Taken together, our results suggest that *Stag2* loss diminishes the number of, but not the strength, short chromosome loops, including promoter-anchored loops. Highly expressed genes might be more reliant on these loops for transcription and are preferentially downregulated by *Stag2* loss (Figure 4—figure supplement 4B-D).

We also performed pile-up analysis of local chromatin regions flanking TSSs (Figure 7F). Strikingly, we observed a clear stripe that extended from the TSS of downregulated gene only in the direction of transcription. The formation of promoter-anchored stripes (P-stripes) on aggregated plots is consistent with one-sided loop extrusion from the promoter to the gene body. The P-stripe was still present in *Stag2^f/y^;Nes^Cre^* cells, suggesting that STAG1 could compensate for the loss of STAG2 and mediate its formation (Figure 7F and Figure 7—figure supplement 3E).

## Discussion

Cohesin is critical for the three-dimensional (3D) organization of the genome by extruding chromosome loops. Acute depletion of cohesin abolishes chromosome loops and TADs, but has moderate effects on transcription. The two forms of cohesin in vertebrate somatic cells, namely STAG1-cohesin and STAG2-cohesin, have largely redundant functions in supporting sister-chromatid cohesion and cell viability, but they have nonredundant functions during development. In this study, we have established a myelination-promoting function of STAG2 in the CNS in the mouse. We further provide evidence linking hypomyelination caused by STAG2 loss to reduced promoter-anchored loops at myelination genes in oligodendrocytes.

### Myelination functions of STAG2 and implications for cohesinopathy

Selective ablation of *Stag2* in the nervous system in the mouse causes growth retardation, neurological defects, and premature death. STAG2 loss delays the maturation of oligodendrocytes and reduces the expression of highly active myelin and cholesterol biosynthesis genes in oligodendrocytes, resulting in hypomyelination in the CNS. Hypomyelination disorders in humans and mice are known to produce abnormal neurological behaviors similar to those seen in our *Stag2* cKO mice, suggesting that hypomyelination is a major underlying cause for the phenotypes in *Stag2* cKO mice. The growth retardation in these mice can be explained by insufficient secretion of growth hormones, which may be a consequence of defective neuronal signaling.

Mutations of cohesin subunits and regulators, including STAG2, cause the Cornelia de Lange syndrome (CdLS) and other similar developmental diseases, collectively termed cohesinopathy. CdLS patients exhibit short stature and developmental defects in multiple tissues and organs, including the brain. Although STAG2 mutations are implicated in human cohesinopathy, these mutations are rare and hypomorphic (Soardi et al., 2017). The cohesin loader NIPBL is the most frequently mutated cohesin regulator in cohesinopathy (Mannini et al., 2013). NIPBL deficiency is expected to affect the functions of both STAG1- and STAG2-cohesin. It is possible that the partial loss of STAG2-cohesin function leads to subtle myelination defects in patients with cohesinopathy. Indeed, lack of myelination in certain brain regions of CdLS patients has been reported (Avagliano et al., 2017; Vuilleumier et al., 2002). As myelination of the CNS mostly occurs after birth and during childhood, strategies aimed at enhancing myelination might help to alleviate certain disease phenotypes and symptoms.

### Mechanisms by which STAG2 promotes myelination

STAG2 promotes oligodendrocyte maturation and the expression of myelination genes in mature oligodendrocytes. Because STAG2 does not have an established cohesin-independent function, it most likely activates the myelination-promoting transcriptional program as a core component of cohesin. Consistent with previous reports (Rao et al., 2017), loss of STAG2-cohesin in oligodendrocytes does not affect genome compartmentalization, but reduces the number of relatively short chromosome loops, including promoter-anchored loops. Promoter-anchored loops at downregulated genes are reduced to a greater extent than those at stable and upregulated genes. These findings suggest that STAG2-cohesin promotes the myelination transcriptional program by forming promoter-anchored loops.

Pile-up analysis of Hi-C maps reveals the formation of asymmetric promoter-anchored stripes in the direction of transcription at downregulated genes, indicative of active loading of cohesin at TSSs followed by one-sided loop extrusion from the promoter to the gene body. The stripes are, however, not reduced in STAG2-deficient cells. Because both forms of cohesin are capable of loop extrusion, it is possible that STAG1-cohesin can compensate for the loss of STAG2-cohesin in loop extrusion. It remains to be tested whether the intrinsic kinetics and processivity of loop extrusion mediated by the two forms of cohesin are differentially regulated by cellular factors or posttranslational modifications and whether these differences contribute to their nonredundant roles in transcription regulation.

We envision three possibilities that may account for why oligodendrocytes, but not other cell types, are more severely affected by *Stag2* loss in the CNS. First, STAG2-cohesin may be more abundant than STAG1-cohesin in postmitotic OLs, making them more dependent on STAG2 for proper functions. Second, STAG1-cohesin preferentially localizes to CTCF-enriched TAD boundaries whereas STAG2-cohesin is more enriched at enhancers lacking CTCF (Kojic et al., 2018). Enhancers are critical for cell-type-specific gene transcriptional programs. To cooperate with the axonal growth during postnatal neurodevelopment, enhancer-enriched transcription factors induce timely and robust gene expression in oligodendrocytes for proper myelination (Mitew et al., 2014). The high demand for enhancer function may render the transcription of myelination genes more reliant on STAG2-cohesin. Finally, the C-terminal regions of STAG1 and STAG2 are divergent in sequence and may bind to different interacting proteins and be subjected to differential regulation. STAG2 may interact with oligodendrocyte-specific transcription factors and be preferentially recruited to myelination genes. It will be interesting to investigate the interactomes of STAG1 and STAG2 in oligodendrocytes using mass spectrometry.

### STAG2-mediated chromosome looping and transcription

**Figure 8.**
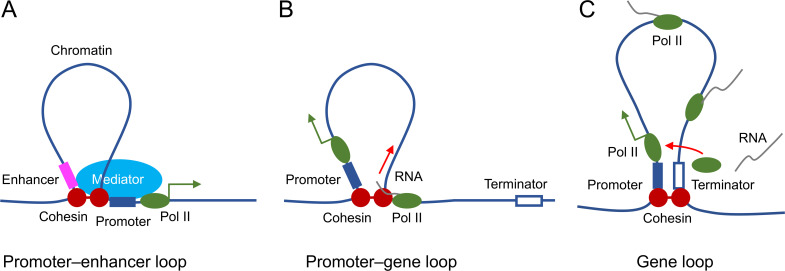
Proposed roles of STAG2-cohesin-mediated loop extrusion during transcription in oligodendrocytes. (**A**) STAG2-cohesin-mediated chromosome looping connects the enhancer and the promoter, thus facilitating interactions among oligodendrocyte-specific transcription factors, the mediator complex, and the general transcription machinery including RNA polymerase II. (**B**) STAG2-cohesin travels along the gene body via transcription-coupled loop extrusion to facilitate pre-mRNA processing. (**C**) STAG2-cohesin mediates the formation of gene loops that bring the terminator close to the promoter and facilitate Pol II recycling for multiple rounds of transcription.

The mechanisms by which STAG2-dependent chromosome looping facilitates transcription are unclear at present. We propose several models that are not mutually exclusive (Figure 8). First, by forming promoter–enhancer loops, STAG2-cohesin brings the mediator complex and other enhancer-binding factors to the spatial proximity of the general transcriptional machinery at the promoter, thereby enhancing RNA polymerase II recruitment and transcription initiation. The existence of P-stripes at STAG2-dependent genes in the Hi-C maps suggests that STAG2-mediated promoter–enhancer loops may involve enhancers located in the gene body. Second, loop extrusion by STAG2-cohesin may promote transcription elongation by regulating transcription-coupled pre-mRNA processing. For example, STAG2 has been shown to interact with RNA–DNA hybrid structures termed R-loops in vitro and in cells (Pan et al., 2020; Porter et al., 2021). R-loops formed between the nascent pre-mRNA and the DNA template impede transcription elongation and need to be suppressed (Moore and Proudfoot, 2009). When traveling with the transcription machinery on DNA, STAG2-cohesin might directly suppress R-loop formation or recruit other factors, such as the spliceosome, for cotranscriptional pre-mRNA processing and R-loop resolution. Third, STAG2-cohesin may establish promoter–terminator gene loops to recycle the RNA polymerase II that has finished one cycle of transcription back to the TSS for another round of transcription. Future experiments using high-resolution Hi-C methods in oligodendrocytes and ChIP-seq experiments with additional enhancer- and promoter-specific histone marks will allow us to better define the nature of STAG2-dependent promoter-anchored loops and stripes. It will also be interesting to examine whether *Stag2* deletion causes the accumulation of R-loops in downregulated genes and the incomplete splicing of their pre-mRNAs.

### Conclusion

We have discovered a requirement for the cohesin subunit STAG2 in the myelination of the CNS in mammals. Our findings implicate hypomyelination as a contributing factor to certain phenotypes of cohesinopathy, including growth retardation and neurological disorders. We provide evidence to suggest that STAG2 promotes the myelination transcriptional program in oligodendrocytes through the formation of promoter-anchored loops. Our study establishes oligodendrocytes as a physiologically relevant cell system for dissecting the cellular functions and regulatory mechanisms of cohesin-mediated chromosome folding and genome organization.

## Materials and methods

### Generation of mouse lines and mouse husbandry

All animals were handled in accordance with institutional guidelines of the Institutional Animal Care and Use Committee (IACUC; AAALAC unit number 000673) of University of Texas (UT) Southwestern Medical Center under the animal protocol number (APN) 102335. The *Stag2* locus was targeted by inserting one neo cassette and two loxP sites flanking exon 8 via homologous recombination in the mouse embryonic stem (ES) cells. G418-selected positive ES clones were screened for successful targeting by nested PCR tests on both 5′ and 3′ integration sites of loxP. Four confirmed ES clones were then microinjected into mouse blastocysts. The chimeras were bred to the R26FLP mouse line for the removal of the neo cassette. *Stag2^f/+^* mice with the 129/B6 background were crossed with *Stag2^f/y^* or WT C57BL/6J mice and maintained on this background. For the generation of the inducible system of *Stag2^f/y^;Rosa26^CreErt2^* mice, *Stag2^f/f^* mice were bred to the mouse strain that contains two alleles of the conditional Cre-ER^T2^ cassette (B6.129-Gt(ROSA)26Sortm1(cre/ERT2)Tyj/J, JAX stock #008463) (Ventura et al., 2007). For the generation of the nervous system-specific *Stag2^f/y^;Nes^Cre^* mice, the *Stag2^f/f^* mice were crossed with the transgenic mice carrying one allele of Cre recombinase driven by the rat nestin promoter and enhancer (Tg(Nes-cre)1Kln, JAX stock #003771) (Giusti et al., 2014; Tronche et al., 1999).

Whole-body knockout mice were generated by CRISPR–Cas9 gene editing technology. Briefly, a pair of guide RNAs (sgRNA; sequences listed in the Key Resource Table) targeting genomic sequence flanking exon 8 of the *Stag2* locus were tested for cutting efficiency in cell culture, transcribed in vitro, purified, checked for integrity, and microinjected into B6C3F1 mouse zygotes along with the *Cas9* mRNA (5-methylcytidine, pseudouridine, TriLink). 20 ng/μl of *Cas9* mRNA and 10 or 20 μg/μl each of sgRNA were used. The injected embryos were transferred to the surrogate mother on the same day. Mosaic F_0_ founders carrying the *Stag2^null^* allele were identified by PCR genotyping. The reduction of the STAG2 protein was confirmed by western blotting in multiple tissues. The F_0_ founders were crossed with WT C57BL/6J mice to generate the *Stag2^+/−^* F_1_. The mutations in F_1_ were identified by Sanger Sequencing. Two mouse lines carrying genomic deletions between the Cas9 cleavage sites were chosen for the generation of *Stag2^null^* mouse embryos.

All mice were housed in the antigen-free barrier facility with 12 hr light/dark cycles (6 AM on and 6 PM off). Mice were fed a standard rodent chow (2016 Teklad Global 16% protein rodent diet, Harlan Laboratories).

### Immunoblotting

The C-terminal fragment of human STAG2 protein was expressed and purified from *Escherichia coli* and used as the antigen to generate rabbit polyclonal antibodies against STAG2 at YenZym. Other antibodies were purchased from the following commercial sources: anti-SMC1 (Bethyl Laboratories, A300-055A), anti-SMC3 (Bethyl Laboratories, A300-060A), anti-RAD21 (Bethyl Laboratories, A300-080A), anti-SA1 (Bethyl Laboratories, A302-579A), anti-SA2 (Bethyl Laboratories, A302-581A), anti-α-TUBULIN (Sigma-Aldrich, DM1A), anti-MBP (Abcam, ab7349), anti-PLP1 (Abcam, ab28486), and anti-H3K27ac (Abcam, ab4729).

For immunoblotting, brain hemispheres were homogenized in a Precellys tissue homogenizer (Bertin Instruments) with the lysis buffer [20 mM Tris–HCl (pH 7.7), 137 mM NaCl, 2 mM Ethylenediamine tetraacetic acid (EDTA), 10% (vol/vol) glycerol, 1% (vol/vol) Triton X-100, 0.5 mM dithiothreitol, 1 mM Phenylmethylsulfonyl fluoride (PMSF), 1 mM Na_3_VO_4_, 10 mM β-glycerophosphate, 5 mM NaF, and protease inhibitors (Roche)]. Homogenized brain tissues were lysed on ice for 1 hr. The lysate was then subjected to centrifugation at 20,817 × *g* at 4°C for 20 min and further cleared by filtering through a 0.45 μm filter. The cleared lysate was analyzed by sodium dodecyl sulfate–polyacrylamide gel electrophoresis and transferred to membranes, which was then incubated with the appropriate primary and secondary antibodies. The blots were imaged with the Odyssey Infrared Imaging System (LI-COR).

### Tissue histology and immunohistochemistry

Mouse brains were fixed in 10% neutral buffered formalin solution for 48 hr followed by paraffin embedding and coronal or sagittal sectioning at 5 μm. H&E staining and LFB staining were performed by the Molecular Pathology Core at UT Southwestern Medical Center. Investigators were blinded to the genotype. Images were acquired with the DM2000 microscope (Leica) at ×1.25 resolution.

Immunohistochemistry was performed as previously described (Choi et al., 2016). Briefly, deparaffinized sections were fixed with 4% paraformaldehyde, subjected to antigen retrieval by boiling with 10 mM sodium citrate (pH 6.0), and then incubated with the indicated antibodies at 1:100 dilution. The slides were scanned with an Axioscan.Z1 microscope (Zeiss) at ×40 resolution at the Whole Brain Microscopy Facility at UT Southwestern Medical Center. Images were processed and quantified with ImageJ. For the myelinated fiber length measurement and coherency analysis, coronal sections of the brain cortex stained with the anti-MBP antibody were processed as previously described (van Tilborg et al., 2017). The myelinated axial thinning and fiber length measurement were performed by the plugin DiameterJ. The coherency analysis of myelinated axons was performed with the plugin OrientationJ.

### Isolation of primary oligodendrocytes

The immunomagnetic isolation of oligodendrocytes from *Stag2^f/y^* and *Stag2^f/y^;Nes^Cre^* P12-P14 pups was conducted using anti-O4 microbeads (Miltenyi Biotec) according to a published protocol (Flores-Obando et al., 2018). Briefly, brain cortices were dissected, pooled, minced into 1 mm^3^ cubes, and incubated with the Papain dissociation solution (neurobasal medium with 1% penicillin–streptomycin, 1% L-glutamine, 2% B27 supplement, 20–30 U/ml of Papain, and 2500 U DNase I) in a 37°C, 5% CO_2_ incubator for more than 20 min. The enzymatic digestion was inactivated by the addition of 1 ml of fetal bovine serum (FBS). Gentle trituration by 10 ml, 5 ml and 1-ml pipettes was applied to break up cell clumps. Cells were collected by centrifugation (200 × *g*, 10 min), washed first with serum-containing medium (neurobasal medium with 1% penicillin–streptomycin, 1% L-glutamine, 2% B27 supplement, and 10% FBS), and then with the magnetic cell sorting (MCS) buffer (phosphate-buffered saline, pH 7.2, with 0.5% bovine serum albumin [BSA], 0.5 mM EDTA, 5 μg/ml insulin and 1 g/l glucose). The cell pellet was resuspended in the MCS buffer and incubated with anti-O4 microbeads at 10 μl/10^7^ cells at 4°C for 15 min followed by 1× wash with the MCS buffer. The O4^+^ immature oligodendrocytes were sorted through the magnetic LS columns according to the manufacturer’s instruction. Freshly prepared oligodendrocytes were directly used or fixed for subsequent analysis.

### Metabolic cage analysis

Mice were singly housed in shoebox-sized cages with a 5-day acclimation period followed with a 4-day recording period. Recorded parameters were analyzed by the TSE system and normalized to body weight. The experiments were conducted by the core personnel under the core protocol at the Metabolic Phenotyping Core at UT Southwestern Medical Center. Investigators were blinded to the genotype.

### Growth hormone and IGF-1 detection

Blood samples were collected from facial bleeding without fasting. Plasma growth hormone levels were determined with the rat/mouse growth hormone ELISA kit (EMD Milipore, EZRMGH-45K). Plasma IGF-1 concentrations were measured using the mouse/rat IGF1 Quantikine ELISA kit (R&D Systems).

### Sterol and oxysterol composition analysis

Brain hemispheres were preweighed and snap-frozen for extraction and measurement by mass spectrometry. The sterol extraction and quantitative analysis were conducted at the Center of Human Nutrition at UT Southwestern Medical Center as described previously (McDonald et al., 2012).

### Electron microscopy

*Stag2^f/y^* and *Stag2^f/y^;Nes^Cre^* P18 pups were transcardially perfused with 4% paraformaldehyde, 1% glutaraldehyde in 0.1 M sodium cacodylate buffer (pH 7.4). Tissues were dissected and fixed with 2.5% (vol/vol) glutaraldehyde in 0.1 M sodium cacodylate buffer (pH 7.4) for at least 2 hr. After three rinses with the 0.1 M sodium cacodylate buffer, optic nerve samples were embedded in 3% agarose and sliced into small blocks. All samples were again rinsed with the 0.1 M sodium cacodylate buffer three times and postfixed with 1% osmium tetroxide and 0.8% potassium ferricyanide in the 0.1 M sodium cacodylate buffer for 3 hr at room temperature. Blocks were rinsed with water and *en bloc* stained with 4% uranyl acetate in 50% ethanol for 2 hr. Samples were dehydrated with increasing concentrations of ethanol, transitioned into propylene oxide, infiltrated with Embed-812 resin, and polymerized in a 60°C oven overnight. Blocks were sectioned with a diamond knife (Diatome) on a Leica Ultracut 7 ultramicrotome (Leica Microsystems) and collected onto copper grids, poststained with 2% aqueous uranyl acetate and lead citrate. Images were acquired on a Tecnai G2 Spirit transmission electron microscope (Thermo Fisher) equipped with a LaB6 source using a voltage of 120 kV. Tissue processing, sectioning, and staining were completed by the Electron Microscopy Core at UT Southwestern Medical Center.

### RNA-seq library preparation and sequencing

Total RNA was extracted from brain hemispheres or isolated oligodendrocytes with Trizol. RNA integrity was determined by the Agilent BioAnalyzer 2100. TruSeq Stranded mRNA library prep kit (Illumina) was used to generate the mRNA libraries. The libraries were analyzed by the Bioanalyzer and multiplexed and sequenced using the NextSeq 500 high output kit (400 M reads) for the brain libraries or NextSeq 500 mid output kit (130 M reads) for the isolated oligodendrocytes libraries at the Next Generation Sequencing Core at UT Southwestern Medical Center.

### Differential expression and pathway analysis

Raw data from the sequencer were demultiplexed and converted to fastq files using bcl2fastq (v2.17, Illumina). The fastq files were checked for quality using fastqc (v0.11.2) Andrews, 2010 and fastq_screen (v0.4.4) (Wingett, 2011). Fastq files were mapped to the mm10 mouse reference genome (from iGenomes) using STAR (Dobin et al., 2013). Read counts were then generated using featureCounts (Liao et al., 2014). TMM normalization and differential expression analysis were performed using edgeR (Robinson et al., 2010). Pathway analysis was performed with the IPA software. Genes with more than 1.5-fold change and false-discovery rate FDR <0.01 were included in the brain RNA-seq pathway analysis. Genes with more than twofold change and FDR <0.05 were used for the pathway analysis of the RNA-seq data from oligodendrocytes.

### RT-qPCR analysis

Single-stranded cDNAs were converted from 2 µg of total RNA extracted from mouse brains with the high-capacity cDNA reverse transcription kit (Applied Biosystems). Quantitative PCR was conducted to determine transcript levels using gene-specific TaqMan probes (Applied Biosystems).

### Single-cell RNA-seq

Single-cell suspension was prepared from forebrains of P13 *Stag2^f/y^* or *Stag2^f/y^;Nes^Cre^* pups using the Papain Dissociation System (Worthington Biochemical, LK003150) according to the manufacturer’s instructions. Biological duplicates were made for each genotype. Single-cell RNA-seq libraries were generated with the Chromium Single Cell 3′ GEM, Library & Gel Bead Kit v3 (10× Genomics) according to the manufacturer’s guidelines. Cell density and viability were checked by the TC-20 Cell Counter (Bio-Rad). Cells were then loaded onto Chip B in the Chromium Controller (10× Genomics). 10,000 cells were targeted for each sample. The libraries were analyzed by the Bioanalyzer (Agilent) and pair-end sequenced in two flowcells of the NextSeq 500 High Output (400 M) run. The sequencing was performed at the Next Generation Sequencing Core at UT Southwestern Medical Center.

Data demultiplexing and alignment were performed using the Cell Ranger pipeline (https://support.10xgenomics.com/single-cell-gene-expression/software/pipelines/latest/using/ mkfastq) (10× Genomics). The raw features, barcodes, and matrixes were used as input for further analysis using the R package Seurat3 (Butler et al., 2018; Stuart et al., 2019) (https://satijalab.org/seurat/). Cells were filtered by the following criteria: nFeature_RNA (200–9500) and percent.mt <10. After filtering, a total of 5834 cells in *Stag2^f/y^#1*, 4699 cells in *Stag2^f/y^#2*, 9050 cells in *Stag2^f/y^;Nes^Cre^#1*, and 3073 cells in *Stag2^f/y^;Nes^Cre^#2* were used for downstream analysis. 2000 variable features were found from each normalized dataset. All datasets were then integrated using identified anchors (dims = 1:30). Standard scaling and principal component analysis, clustering (resolution = 0.5), and tSNE reduction (dims = 1:30) were performed on the integrated dataset. Cluster biomarkers were identified, and top features were examined. Clusters were then manually assigned to distinct cell-type identities with knowledge from previous studies (Cahoy et al., 2008; Dulken et al., 2019; Marques et al., 2018; Marques et al., 2016; Marton et al., 2019; Saunders et al., 2018; Zeisel et al., 2018; Zywitza et al., 2018) (http://www.brainrnaseq.org/) (http://dropviz.org/). Clusters with the same cell-type identities were merged. Five clusters of oligodendrocyte lineage [cycling oligodendrocyte progenitors (OPCcycs), oligodendrocyte progenitors (OPCs), newly formed oligodendrocytes (NFOLs), myelin-forming oligodendrocytes (mFOLs), and fully matured oligodendrocytes (MFOLs)] were identified and selected for indicated gene expression comparison and plotting using Vlnplot or FeaturePlot functions. The trajectory analysis was performed using Monocle3 (Cao et al., 2019) in the oligodendrocyte cell population. Gene density plot over pseudotime was generated as previously described (Luecken and Theis, 2019).

### ChIP-seq

Chromatin immunoprecipitation (ChIP) was performed as previously described (Liu et al., 2017). Briefly, isolated oligodendrocytes were fixed with 1% formaldehyde and fragmented with a sonicator (Branson 450). The fragmented chromatin was incubated with antibodies overnight at 4°C. Dynabeads Protein A (Thermo Fisher Scientific) was used for the immunoprecipitation. Libraries were generated by the Next Gen DNA Library Kit (Active Motif) with the Next Gen Indexing Kit (Active Motif) for STAG2 ChIP-seq or the KAPA HyperPrep Kits (KAPA Systems) for histone ChIP-seq. The libraries were analyzed by the Bioanalyzer and pool sequenced with the NextSeq 500 mid output (130 M) kit. After mapping reads to the mouse genome (mm10) by bowtie2 (v2.2.3) (Langmead and Salzberg, 2012) with the parameter ‘–sensitive’, we performed filtering by removing alignments with mapping quality less than 10 and then removing duplicate reads identified by Picard MarkDuplicates (v1.127). For STAG2 ChIP-seq, Picard MarkDuplicate was used to remove duplicates together with options to use molecular identifiers (MIDs) information in the reads. Enriched regions (peaks) were identified using MACS2 (v2.0.10) (Zhang et al., 2008), with a *q*-value cutoff of 0.05 for peaks. Peak regions were annotated by HOMER (Ross-Innes et al., 2012).

### Hi-C library generation, sequencing, and analysis

Hi-C was performed at the Genome Technology Center at NYU Langone Health from 3.5 to 4.0 μg of DNA isolated from cells cross-linked with 2% formaldehyde at room temperature for 10 min. Experiments were performed in duplicates following the instructions from the Arima Hi-C kit (Arima Genomics, San Diego, CA). Subsequently, Illumina-compatible sequencing libraries were prepared by using a modified version of the KAPA HyperPrep library kit (KAPA BioSystems, Willmington, MA). Quality check steps were performed to assess the fraction of proximally ligated DNA labeled with biotin, and the optimal number of PCR reactions needed to make libraries. The libraries were loaded into an Illumina flowcell (Illumina, San Diego, CA) on a NovaSeq 6000 instrument for paired-end 50 reads.

Hi-C analysis was performed using the HiC-Bench pipeline (Lazaris et al., 2017; Tsirigos et al., 2012) (https://github.com/NYU-BFX/hic-bench) and HiC-Pro v3.1.0 (Servant et al., 2015). The read pairs were aligned and filtered with the following parameters: Genome-build=mm10; –very-sensitive-local –local; mapq = 20; –min-dist 25000 –max-offset 500. The Juicer ‘pre’ tool (Durand et al., 2016) (RRID: SCR_017226, v1.11.09; https://github.com/aidenlab/juicer) was used to generate the.hic file with default parameters. Sample duplicates were combined. The compartment analysis was done using the HOMER tool (Heinz et al., 2010) (http://homer.ucsd.edu/homer/index.html) with 100 kb bins. H3K27ac ChIP-seq data were used to assign A/B compartments. Eigenvector-1 bins were considered shifted (AB and BA) when the bin sign changed and the delta value was greater than 1.5. TADs and boundaries were identified at 40 kb resolution with the HiCRatio method with the follow parameters: –min-lambda=0.0 –max-lambda=1.0 –n-lambda=6 –gamma = 0 –distance = 500 kb –fdr = 0.1. TADs were also identified using the Juicer tools (v1.22.01) arrowhead at 10 and 25 kb resolution. Aggregate TAD analysis was performed on TAD boundaries by coolpup.py (Flyamer et al., 2020) or GENOVA (van der Weide et al., 2021). The.hic files were converted to.cool format for visualization and plotting with pyGenomeTracks (Lopez-Delisle et al., 2021) at 5 kb resolution.

### Loop analysis and RNA-seq integration

The loops were classified into group-specific loops and common loops by using the significance cutoffs provided by Fit-HiC (Ay et al., 2014). A *q*-value cutoff of 0.01 was used to identify significant loops in both groups. A loop is considered ‘group-specific’ if it is only present in one group with a *q* value <0.01 and not present in the other group with cutoff of *q* val <0.1. Loop anchors were annotated with the gene promoter information (promoter defined as ±2 kb from the TSS). The genes were classified into ‘down’ and ‘up’ regulated genes using an FDR cutoff of 0.05, logFC cutoff of ±0.58 and logCPM >0. ‘stable’ or less changed genes are defined as logFC <0.38, and logCPM >0. Random 1000 genes were chosen for analysis and plotting. The active genes (logCPM >0) were also grouped in ‘high’, ‘mid’, and ‘low’ expression groups by separating the genes in three quantiles according to the logCPM values. For the loop enrichment scores, normalized contact scores were computed using Fit-HiC at 10 kb resolution and bias corrected. Pile-up analysis was performed with coolpup.py (Flyamer et al., 2020) with the KR method to balance the weight and random shift controls for distance normalization at 5 kb or using GENOVA.

## Data Availability

The RNA-seq, scRNA-seq, ChIP-seq, and Hi-C datasets generated and analyzed during the current study are available in the GEO repository, with the accession number GSE186894. The following dataset was generated: ChengN KanchwalaM EversBM XingC YuH 2021STAG2 promotes the myelination transcriptional program in oligodendrocytesNCBI Gene Expression OmnibusGSE18689410.7554/eLife.77848PMC943967935959892

## Additional files

Supplementary file 1. List of enriched pathways of differentially expressed genes between wild-type (WT) and *Stag2* KO mouse brains as revealed by ingenuity pathway analysis (IPA).

Supplementary file 2. List of differentially expressed genes between wild-type (WT) and *Stag2* KO oligodendrocytes, with the status of STAG2 binding at their promoters and the numbers of promoter-anchored loops indicated.

Transparent reporting form

## Appendix 1

**Appendix 1—key resources table:**

Reagent type (species) or resource	Designation	Source or reference	Identifiers	Additional information
Strain, strain background (*Mus musculus*, female)	*Stag2^+/−^*	This paper		Exon8 of *Stag2* was targeted by CRISPR–Cas9 (see Materials and methods)
Strain, strain background (*Mus. musculus*, both sex)	*Stag2^f/y^*; *Stag2^f/f^*	This paper		Exon8 of *Stag2* genomic locus was flanked by loxP sites (see Materials and methods)
Strain, strain background (*Mus. musculus*, both sex)	C57BL/6J	The Jackson Laboratory	000664; RRID:IMSR_JAX:000664	
Strain, strain background (*Mus. musculus*, both sex)	B6.129-Gt(ROSA)26 Sortm1(cre/ERT2)Tyj/J	The Jackson Laboratory	008463; RRID:IMSR_JAX:008463	
Strain, strain background (*Mus. musculus*, male)	B6.Cg-Tg(Nes-cre)1Kln/J	The Jackson Laboratory	003771; RRID:IMSR_JAX:003771	
Antibody	anti-STAG2 (Rabbit polyclonal)	This paper		The C-terminus recombinant protein of STAG2 (*Homo sapiens*) was used to generate the antibody; WB (1:1000)
Antibody	anti-α-TUBULIN (Mouse monoclonal)	Sigma-Aldrich	T9026; RRID:AB_477593	WB (1:1000)
Antibody	anti-SA1 (Rabbit polyclonal)	Bethyl Laboratories	A302-579A; RRID:AB_2034857	WB (1:1000)
Antibody	anti-SMC1 (Rabbit polyclonal)	Bethyl Laboratories	A300-055A RRID:AB_2192467	WB (1:1000)
Antibody	anti-SMC3 (Rabbit polyclonal)	Bethyl Laboratories	A300-060A; RRID:AB_67579	WB (1:1000)
Antibody	anti-RAD21 (Rabbit polyclonal)	Bethyl Laboratories	A300-080a; RRID:AB_2176615	WB (1:1000)
Antibody	anti-MBP (Rat monoclonal)	Abcam	ab7349; RRID:AB_305869	IHC (1:100)
Antibody	anti-PLP1 (Rabbit polyclonal)	Abcam	ab28486; RRID:AB_776593	IHC (1:100)
Antibody	anti-GFAP (Rabbit polyclonal)	Abcam	ab7260; RRID:AB_305808	IHC (1:100)
Antibody	anti-MAP2 (Rabbit polyclonal)	Abcam	ab32454; RRID:AB_776174	IHC (1:50)
Antibody	anti-H3K27ac (Rabbit polyclonal)	Abcam	ab4729; RRID:AB_2118291	ChIP (5 μl per test)
Antibody	anti-O4 Microbeads (Mouse monoclonal)	Miltenyi Biotec	130-094-543; RRID:AB_2847907	MACS (10 μl per 10^7^ cells)
Antibody	anti-rabbit IgG (H+L), DyLight 800 Conjugate (Goat polyclonal)	Cell Signaling Technology	5151 S; RRID:AB_10697505	WB (1:5000)
Antibody	anti-mouse IgG (H+L), DyLight 680 Conjugate (Goat polyclonal)	Cell Signaling Technology	5470 S; AB_10696895	WB (1:5000)
Antibody	anti-rat IgG (H+L), Alexa Fluor 568 (Goat polyclonal)	Thermo Fisher Scientific	A-11077; RRID:AB_2534121	IHC (1:500)
Antibody	anti-rabbit IgG (H+L), Alexa Fluor 488 (Goat polyclonal)	Thermo Fisher Scientific	A-11008; RRID:AB_143165	IHC(1:500)
Sequence-based reagent	sgRNA#1 target on *Stag2*	This paper	CRISPR single-guide RNA target sequence	Target sequence: TAGCCAACCTCTTTCTCTATTGG
Sequence-based reagent	sgRNA#2 target on *Stag2*	This paper	CRISPR single-guide RNA target sequence	Target sequence: CAGACAGTATACTGTAATGGAGG
Sequence-based reagent	TaqMan probes: *Stag2*	Thermo Fisher Scientific	Mm01311611_m1	
Sequence-based reagent	TaqMan probes: *Klk6*	Thermo Fisher Scientific	Mm00478322_m1	
Sequence-based reagent	TaqMan probes: *Ninj2*	Thermo Fisher Scientific	Mm00450216_m1	
Sequence-based reagent	TaqMan probes: *Cpm*	Thermo Fisher Scientific	Mm01250802_m1	
Sequence-based reagent	TaqMan probes: *Fa2h*	Thermo Fisher Scientific	Mm00626259_m1	
Sequence-based reagent	TaqMan probes: *Gapdh*	Thermo Fisher Scientific	Mm99999915_g1	
Sequence-based reagent	Stag2 gt 5 F	This paper	Genotype sequence primers	GGTATTTACTTGATAGCCAACC
Sequence-based reagent	Stag2 gt 5 R	This paper	Genotype sequence primers	CTCATCTTGATTTTCCTGAAGC
Sequence-based reagent	Stag2 gt 3 F	This paper	Genotype sequence primers	GGTTGAGACAGACAGTATAC
Sequence-based reagent	Stag2 gt 3 R	This paper	Genotype sequence primers	AGGCTGGACTATGACAACTC
Sequence-based reagent	ISH Probe Stag2 P1 F	This paper	Riboprobe synthesis primers	TACGGTACCGACCTTTCAGATGTCACTCCG
Sequence-based reagent	ISH Probe Stag2 P1 R	This paper	Riboprobe synthesis primers	GAAGGATCCGCATCGGATAGACACTCATGA
Sequence-based reagent	ISH Probe Stag2 P2 F	This paper	Riboprobe synthesis primers	TACGGATCCGACCTTTCAGATGTCACTCCG
Sequence-based reagent	ISH Probe Stag2 P2 R	This paper	Riboprobe synthesis primers	GAAGGTACCGCATCGGATAGACACTCATGA
Sequence-based reagent	ISH Probe Stag1 P1 F	This paper	Riboprobe synthesis primers	TTAGGTACCTTACAATGCCTGGTCCTCAGT
Sequence-based reagent	ISH Probe Stag1 P1 R	This paper	Riboprobe synthesis primers	GAAGGATCCCTTTCATTGGCTCTCTTCCC
Sequence-based reagent	ISH Probe Stag1 P2 F	This paper	Riboprobe synthesis primers	TTAGGATCCTTACAATGCCTGGTCCTCAGT
Sequence-based reagent	ISH Probe Stag1 P2 R	This paper	Riboprobe synthesis primers	GAAGGTACCCTTTCATTGGCTCTCTTCCC
Commercial assay or kit	Arima-HiC Kit	Arima Genomics	510008	
Chemical compound, drug	Tamoxifen	Sigma-Aldrich	T5648	
Chemical compound, drug	4-Hydroxytamoxifen	Sigma-Aldrich	H7904	
Software, algorithm	GraphPad Prism	GraphPad Software	RRID:SCR_002798; https://www.graphpad.com/scientific-software/prism/	
Software, algorithm	ImageJ (Fiji)	ImageJ	RRID:SCR_002285; https://imagej.net/software/fiji/	
Software, algorithm	RStudio	The R Foundation	RRID:SCR_000432; https://www.rstudio.com/	
Software, algorithm	Bcl2fastq	Illumina	RRID:SCR_015058	v2.17
Software, algorithm	Fastqc	Andrews, 2010; PMID:24501021	RRID:SCR_014583	v0.11.2
Software, algorithm	Fastq_screen	Wingett, 2011	RRID:SCR_000141; https://www.bioinformatics.babraham.ac.uk/projects/fastqc/	v0.4.4
Software, algorithm	STAR	Dobin et al., 2013; PMID:23104886	RRID:SCR_004463; https://github.com/alexdobin/STAR	v2.5.3a
Software, algorithm	FeatureCounts	Liao et al., 2014; PMID:24227677	RRID:SCR_012919; https://bioconductor.org/packages/release/bioc/html/Rsubread.html	
Software, algorithm	edgeR	Robinson et al., 2010; PMID:19910308	RRID:SCR_012802; https://bioconductor.org/packages/release/bioc/html/edgeR.html	
Software, algorithm	Ingenuity pathway analysis	QIAGEN, Krämer et al., 2014; PMID:24336805	RRID:SCR_008653; https://www.qiagenbioinformatics.com/products/ingenuity-pathway-analysis	
Software, algorithm	MACS2	Zhang et al., 2008; PMID:18798982	RRID:SCR_013291	v2.0.10
Software, algorithm	Bowtie2	Langmead and Salzberg, 2012; PMID:22388286	RRID:SCR_016368	v2.2.3
Software, algorithm	Picard MarkDuplicates	Broad Institute, GitHub Repository	RRID:SCR_006525; http://broadinstitute.github.io/picard/	v1.127
Software, algorithm	HOMER	Heinz et al., 2010, Ross-Innes et al., 2012; PMID:20513432	RRID:SCR_010881; http://homer.ucsd.edu/homer/	
Software, algorithm	Deeptools	Ramírez et al., 2016; PMID:27079975	RRID:SCR_016366; https://deeptools.readthedocs.io/en/develop/	
Software, algorithm	Galaxy	Afgan et al., 2018; PMID:29790989	RRID:SCR_006281; https://usegalaxy.org	
Software, algorithm	Cell Ranger	10× Genomics	RRID:SCR_017344; https://support.10xgenomics.com/single-cell-gene-expression/software/pipelines/latest/using/mkfastq	
Software, algorithm	Seurat	New York Genome Center; Stuart et al., 2019; PMID:31178118	RRID:SCR_016341; https://satijalab.org/seurat	Satija Lab
Software, algorithm	Monocle3	UW Genome Sciences; Cao et al., 2019; PMID:30787437	RRID:SCR_018685; https://cole-trapnell-lab.github.io/monocle3/	Cole Trapnell’s Lab, v3.0
Software, algorithm	HiC-Bench pipeline	Lazaris et al., 2017, Tsirigos et al., 2012; PMID:22113082	https://github.com/NYU-BFX/hic-bench	v0.1
Software, algorithm	Juicer ‘pre’ tool	Durand et al., 2016; PMID:27467249	RRID:SCR_017226; https://github.com/aidenlab/juicer	Aiden Lab, v1.11.09
Software, algorithm	Juicebox	Aiden Lab, BCM	RRID:SCR_021172; https://github.com/aidenlab/Juicebox	v1.5.1
Software, algorithm	Hic2cool	Abdennur and Mirny, 2020; PMID:31290943	https://github.com/4dn-dcic/hic2cool	v0.8.3
Software, algorithm	pyGenomeTracks	Lopez-Delisle et al., 2021; PMID:32745185	https://github.com/deeptools/pyGenomeTracks	v3.7
Software, algorithm	Fit-HiC	Ay et al., 2014; PMID:24501021	https://github.com/ay-lab/fithic	v2.0.7
Software, algorithm	Coolpup.py	Flyamer et al., 2020; PMID:32003791	https://github.com/open2c/coolpuppy	v0.9.5
Software, algorithm	clusterProfiler	Bioinformatics Group, Southern Medical University; Wu et al., 2021; PMID:34557778	RRID:SCR_016884; https://github.com/YuLab-SMU/clusterProfiler	v4.4.1
Software, algorithm	HiC-Pro	Servant et al., 2015; PMID:26619908	RRID:SCR_017643	v3.1.0
Software, algorithm	HiCRep	Yang et al., 2017; PMID:28855260	https://github.com/TaoYang-dev/hicrep	v1.11.0
Software, algorithm	GENOVA	van der Weide et al., 2021; PMID:34046591	https://github.com/robinweide/GENOVA	v1.0.0.9
