# Supplementary figures and images for: STAG2 promotes the myelination transcriptional program in oligodendrocytes
Source: eLife. 2022 Aug 12;11:e77848. doi: 10.7554/eLife.77848 (PMC9439679; doi:10.7554/eLife.77848)

**Figure 1B**

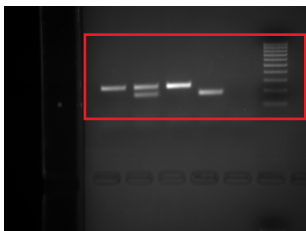

**Figure 1C**

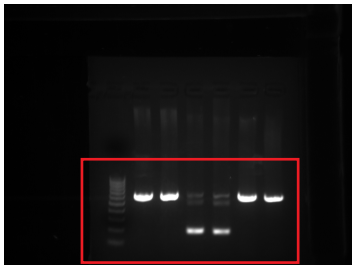

**Figure 1D**

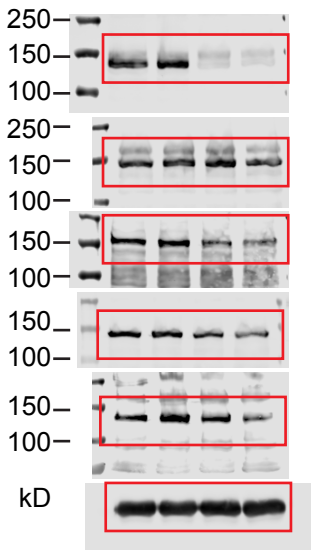

**Figure 1D**

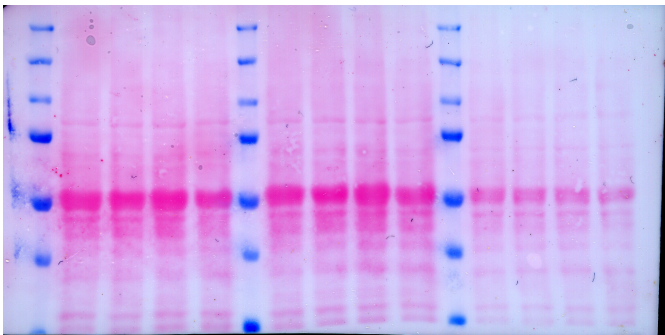

Ponceau S

Supplement: Figure 1—source data 1. [file elife-77848-fig1-data1.pdf]

**Figure 1—  
Figure Supplement 2B**

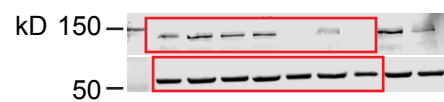

**Figure 1—  
Figure Supplement 2C**

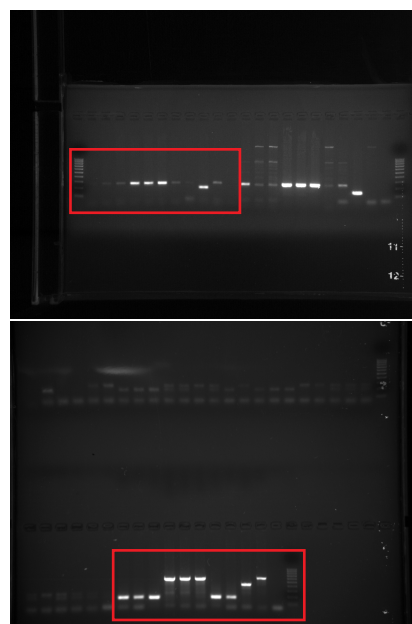

Supplement: Figure 1—figure supplement 2—source data 1. [file elife-77848-fig1-figsupp2-data1.pdf]
